# Diagnostic and Monitoring Strategies for VEXAS Syndrome: Evaluating Sanger Sequencing, NGS, and the SWIM-Score

**DOI:** 10.1007/s10875-025-01932-9

**Published:** 2025-09-30

**Authors:** Lasse von Bornemann Fløe, Kirstine Overgaard Dyrmose, Camilla Darum Sørensen, Maja Nørgaard, Fie Kirstine Udby Pedersen, Johan Vad-Nielsen, Michael Knudsen, Mette Christiansen, Marie Bill, Mads Nyhuus Bendix Rasch, Ellen Margrethe Hauge, Anne Troldborg, Nicklas Heine Staunstrup, Jens Magnus Bernth Jensen

**Affiliations:** 1 Department of Clinical Immunology, Aarhus University Hospital, Aarhus, Denmark; 2 Department of Molecular Medicine, Aarhus University Hospital, Brendstrupgaardsvej 21A, 8200 N Aarhus, Denmark; 3 Department of Hematology, Aarhus University Hospital, Aarhus, Denmark; 4 Department of Rheumatology, Aarhus University Hospital, Aarhus, Denmark; 5 Department of Clinical Medicine, Aarhus University, Aarhus, Denmark; 6 Department of Biomedicine, Aarhus University, Aarhus, Denmark

## Abstract

VEXAS syndrome is an adult-onset autoinflammatory disorder caused by somatic *UBA1* variants, but there are no standardized criteria for genetic testing or diagnostics. This study compared Sanger sequencing and next-generation sequencing (NGS) for detecting *UBA1* variants in patients with suspected VEXAS, assessed the ability of Sanger sequencing to estimate variant allele fractions (VAFs), and evaluated the Maeda et al. scoring system for selecting patients for genetic testing in a primary cohort and a validation cohort. In the primary cohort of 104 patients, Sanger sequencing identified VEXAS variants in 12%, with no additional cases detected by NGS. Sanger sequencing accurately quantified VAFs ranging from 0.1 to 0.9. In a small longitudinal subset (*n* = 3), VAFs in blood correlated with CRP levels, increased over time despite various treatments, but decreased in two patients after initiation of Azacitidine treatment. The novel parameters, VAF in myeloid cells and VEXAS cell concentration, showed promise as exploratory markers for patient monitoring. The Maeda-score, requiring a threshold score of 2 for 100% sensitivity, exhibited low specificity—29% in the primary cohort and 41% in the validation cohort (*n* = 62, with 2 carrying VEXAS variants). In contrast, the simplified SWIM-score—based on Skin involvement, Weight loss, Inflammation, and Macrocytic anemia—achieved 100% sensitivity in both cohorts, with higher specificities of 47% and 65%, respectively. In conclusion, Sanger sequencing reliably detected *UBA1* variants and quantified VAFs. Monitoring VAF and VEXAS cell concentration may track disease progression, and the SWIM-score demonstrated potential for accurately selecting patients for *UBA1* testing.

**Supplementary Information:**

The online version contains supplementary material available at 10.1007/s10875-025-01932-9.

## Introduction

VEXAS syndrome (Vacuoles, E1 enzyme, X-linked, Autoinflammation, Somatic) is an adult-onset systemic autoinflammatory disease caused by somatic variants in the *UBA1* gene located on the X chromosome [1]. This syndrome presents as a phenocopy of inborn errors of immunity, distinguishing itself through the presence of acquired genetic variants in contrast to congenital forms [2]. Currently, there is no established diagnostic standard for VEXAS, and the clinical criteria for testing remain undefined.

Clinically, VEXAS is heterogeneous, presenting with various inflammatory manifestations such as fever, neutrophilic dermatosis, polychondritis, pulmonary infiltrates, and arthritis [1, 3–7]. Hematological disruptions resembling myelodysplastic syndrome, including macrocytic anemia, thrombocytopenia, and bone marrow vacuolization, are also common. VEXAS primarily affects older males [1, 3–7], with an estimated prevalence of approximately 1 in 4,000 to 5,000 in males over 50 years of age [8].

The uncertainty regarding which clinical findings should prompt testing for VEXAS highlights the need for tools to guide the selection process and promote more rational diagnostic practices. Maeda et al. proposed a scoring system based on age at onset above 50 years, cutaneous lesions, pulmonary involvement, chondritis, and macrocytic anemia [9], but its validity in independent cohorts has been questioned [10].

The affected gene, *UBA1*, encodes the protein ubiquitin-like modifier-activating enzyme 1 (E1) [11, 12]. E1 initiates ubiquitylation [13], a pivotal process in numerous cellular functions, including protein degradation, signal transduction, and DNA repair [14]. Humans produce two E1 isoforms: UBA1a and UBA1b [1, 15]. UBA1a is translated from codon 1 and is predominantly nuclear whereas UBA1b is translated from codon 41 and is mainly cytosolic [16]. VEXAS patients typically exhibit variants in codon 41 of *UBA1*, leading to an amino acid substitution in UBA1a, disruption of UBA1b expression, and instead the expression of a truncated isoform, UBA1c, with reduced catalytic activity [1]. These somatic variants are restricted to hematopoietic stem cells and peripheral blood myeloid cells, which exhibit an inflammatory gene-expression profile, thought to drive the disease [1].

**Fig. 1:**
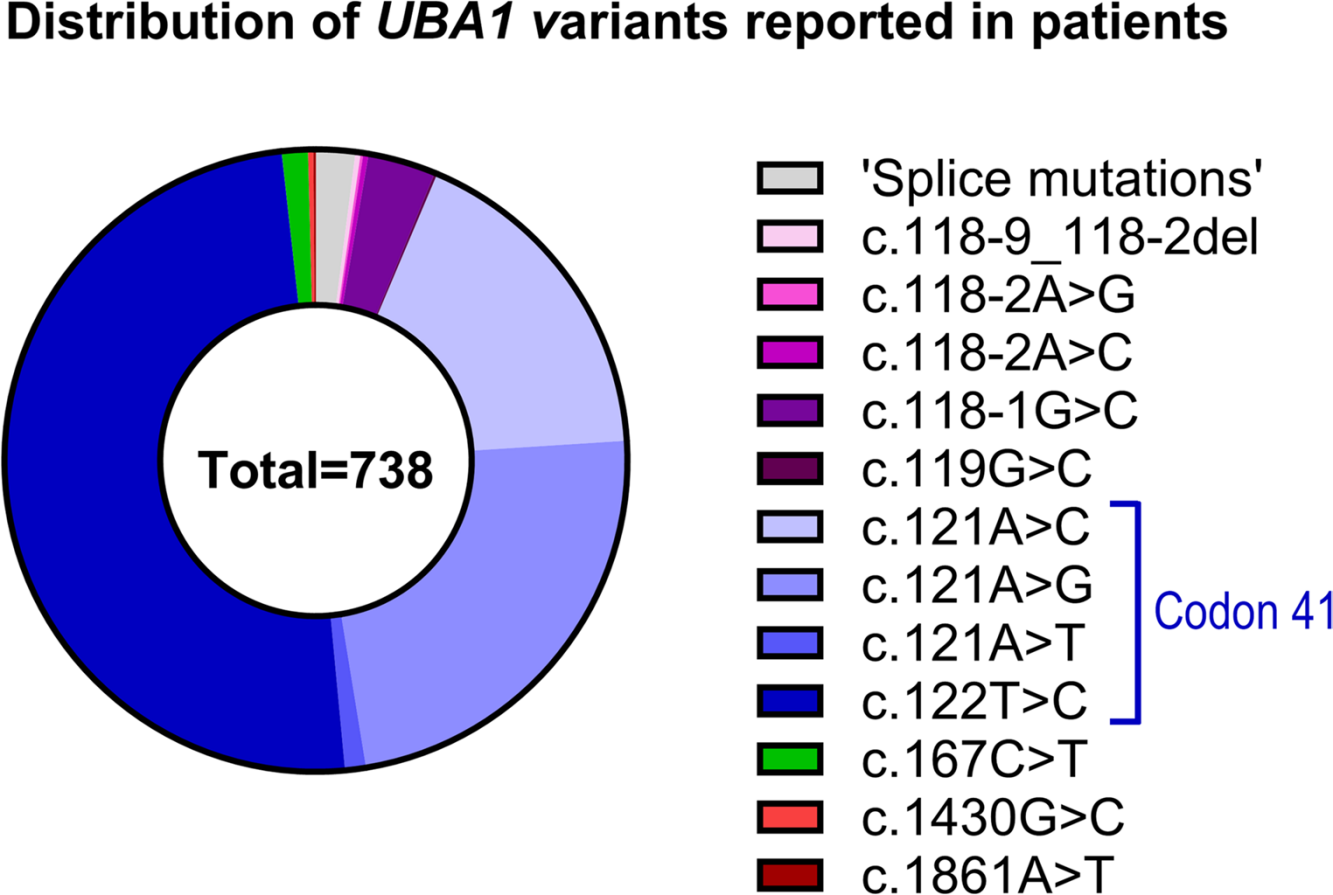
Distribution of specific *UBA1* variants in reported patients with VEXAS (2020–2023) Donut chart illustrating the distribution of specific *UBA1* variants associated with VEXAS in unique patients identified through a literature search. The search included all articles available on PubMed containing the keyword “VEXAS” up to the end of 2023. The included studies are listed in the Table S1

A limited number of *UBA1* variants outside codon 41 have been linked to VEXAS. These variants predominantly affect a neighboring canonical acceptor splice site [17, 18]. Additionally, a few patients are identified with missense loss-of-function variants further 3’ of codon 41 [8, 17, 19]. Overall, in approximately 98% of reported patients, the variants occur within codon 41 of *UBA1* or the adjacent splice site (Fig. 1).

The variant allele fraction (VAF) of *UBA1* in blood (VAF_*Blood*_) varies widely among patients and might not correlate with disease severity [20–22], underscoring the need for novel markers. However, monitoring VAF_*Blood*_ over time in individual patients can be useful for tracking disease progression [7, 23]. Most patients have a VAF_*Blood*_ above 10%, which is detectable by Sanger sequencing [24]. However, some patients present with lower VAF_*Blood*_ [8, 9, 25–27], raising concerns about potential false-negative results with Sanger sequencing compared to more sensitive technologies like NGS. Despite its limitations, Sanger sequencing is cost-effective, rapid, and widely accessible, and can enable quantification of *UBA1* VAFs [5, 27, 28]. Therefore, a performance comparison of *UBA1* variant identification and VAF quantification between NGS and Sanger sequencing is highly desirable.

Our aims were to determine if amplicon-based NGS improves *UBA1* variant identification compared with Sanger sequencing in patients with suspected VEXAS, evaluate Sanger sequencing for estimating *UBA1* VAFs, and assess the Maeda scoring system and an optimized algorithm for selecting patients for *UBA1* testing.

## Methods

### Participants

Participants were individuals suspected of VEXAS and tested for *UBA1* variants at the Department of Clinical Immunology, Aarhus University Hospital, Denmark. Two cohorts were defined: the primary cohort included all individuals tested as of October 26, 2023, and the validation cohort included those tested between October 27, 2023, and November 20, 2024. Data were collected using predefined case-report forms in the REDCap database (Vanderbilt University, Nashville, TN, USA), as detailed in the Supplementary Information.

### DNA Sequencing

DNA was extracted from ethylenediaminetetraacetic acid-stabilized peripheral blood or formaldehyde-fixed, paraffin-embedded bone marrow biopsies, subjected to PCR, and sequenced using standard procedures, as detailed in the Supplementary Information.

### Sequence Analysis

#### Sanger Sequencing

Data were analyzed in Geneious Prime 2021.0.1 (Dotmatics, Boston, MA, USA) using *UBA1* hg38 as reference. VAF_*Blood*_ was estimated with EditR version 10 using default settings [29]. The ‘protospacer’ of maximum length was defined to center the variant position, ensuring an equal number of nucleotides on each side.

#### NGS

Amplicon-based NGS was used to analyze *UBA1* specifically and did not include other genes, as no multigene panel was applied. FASTQ-files were mapped to the hg38 reference genome using BWA-MEM [30]. BAM files were processed with samtools mpileup [31]. VarScan was used to generate VCF files, mpileup2snp for single nucleotide variants and mpileup2indel for insertions and deletions [32], with a variant frequency threshold of 0.001. VCF files were analyzed with VarSeq version 2.4.0 (Golden Helix, Bozeman, MT, USA).

Variants were defined relative to the MANE transcript of *UBA1*, NM_003334.4.

#### VAF Estimation in Myeloid Cells

To estimate the VAF specifically in the blood myeloid cells (VAF_*Myeloid*_), the VAF_*Blood*_ was adjusted based on the fractional contribution of lymphocytes to leukocytes in concurrently collected samples. Blood leukocyte concentrations were determined using Sysmex hematology analyzers.

Since myeloid cells and lymphocytes comprise nearly all nucleated (i.e., genome-containing) cells in the peripheral blood of healthy individuals, the myeloid fraction was estimated as follows:

$$\:\mathrm{M}\mathrm{y}\mathrm{e}\mathrm{l}\mathrm{o}\mathrm{i}\mathrm{d}\:\mathrm{f}\mathrm{r}\mathrm{a}\mathrm{c}\mathrm{t}\mathrm{i}\mathrm{o}\mathrm{n}=\:\frac{\mathrm{T}\mathrm{o}\mathrm{t}\mathrm{a}\mathrm{l}\:\mathrm{l}\mathrm{e}\mathrm{u}\mathrm{k}\mathrm{o}\mathrm{c}\mathrm{y}\mathrm{t}\mathrm{e}\mathrm{s}-\mathrm{T}\mathrm{o}\mathrm{t}\mathrm{a}\mathrm{l}\:\mathrm{l}\mathrm{y}\mathrm{m}\mathrm{p}\mathrm{h}\mathrm{o}\mathrm{c}\mathrm{y}\mathrm{t}\mathrm{e}\mathrm{s}}{\mathrm{T}\mathrm{o}\mathrm{t}\mathrm{a}\mathrm{l}\:\mathrm{l}\mathrm{e}\mathrm{u}\mathrm{k}\mathrm{o}\mathrm{c}\mathrm{y}\mathrm{t}\mathrm{e}\mathrm{s}}$$

VAF_*Myeloid*_ was estimated under the assumption that myeloid cells are the sole carriers of VEXAS-associated variants in peripheral blood, as follows:

$$\:{\mathrm{V}\mathrm{A}\mathrm{F}}_{Myeloid}=\frac{{\mathrm{V}\mathrm{A}\mathrm{F}}_{Blood}}{\mathrm{M}\mathrm{y}\mathrm{e}\mathrm{l}\mathrm{o}\mathrm{i}\mathrm{d}\:\mathrm{f}\mathrm{r}\mathrm{a}\mathrm{c}\mathrm{t}\mathrm{i}\mathrm{o}\mathrm{n}}$$

#### VEXAS Cell Estimation in Peripheral Blood

VEXAS cell concentration was estimated by multiplying the VAF_*Blood*_ by the total leukocyte concentration measured in peripheral blood. This calculation provides an approximation of the absolute number of circulating leukocytes harboring the *UBA1* variant.

### Statistics

C-reactive protein (CRP) measurements below the quantitative range were assigned the threshold value of 4 mg/L. Odds ratios (OR) were calculated using univariate logistic regression, with *UBA1* variant status as the exposure variable. For this exploratory study on a disease without established covariates and limited statistical power, we did not perform covariate analysis to avoid overfitting and potential multicollinearity issues. Fisher’s exact test was applied for group comparisons when the OR was zero or undefined due to a zero denominator. Analyses were conducted in R version 4.4.1. Median difference with 95% confidence intervals (CI) were estimated from 5000 bootstrap samples using Estimation Stats [33]. Linear regression analyses, Receiver Operating Characteristic (ROC) curve analyses, and figure preparation were done in GraphPad Prism version 10.2.3 (Dotmatics, Boston, MA, USA). Matthew’s correlation coefficient (MCC) was calculated as previously described [34].

The statistical significance level was set at 0.05. We did not correct for multiple comparisons due to the exploratory nature of the study, to avoid type II errors. To minimize the risk of Type I errors, we restricted the number of tests to those that were strictly relevant and evaluated the scoring systems in two independent cohorts.

### Ethics

The study was conducted as a quality control project with permission from the board of directors at Aarhus University Hospital, in accordance with Danish legal regulations. This approval included a waiver for obtaining informed consent, limited to relevant health information collected from this hospital within the past five years. Written informed consent was obtained from participants whose VAFs and medication data are presented.

## Results

### Participants

**Fig. 2:**
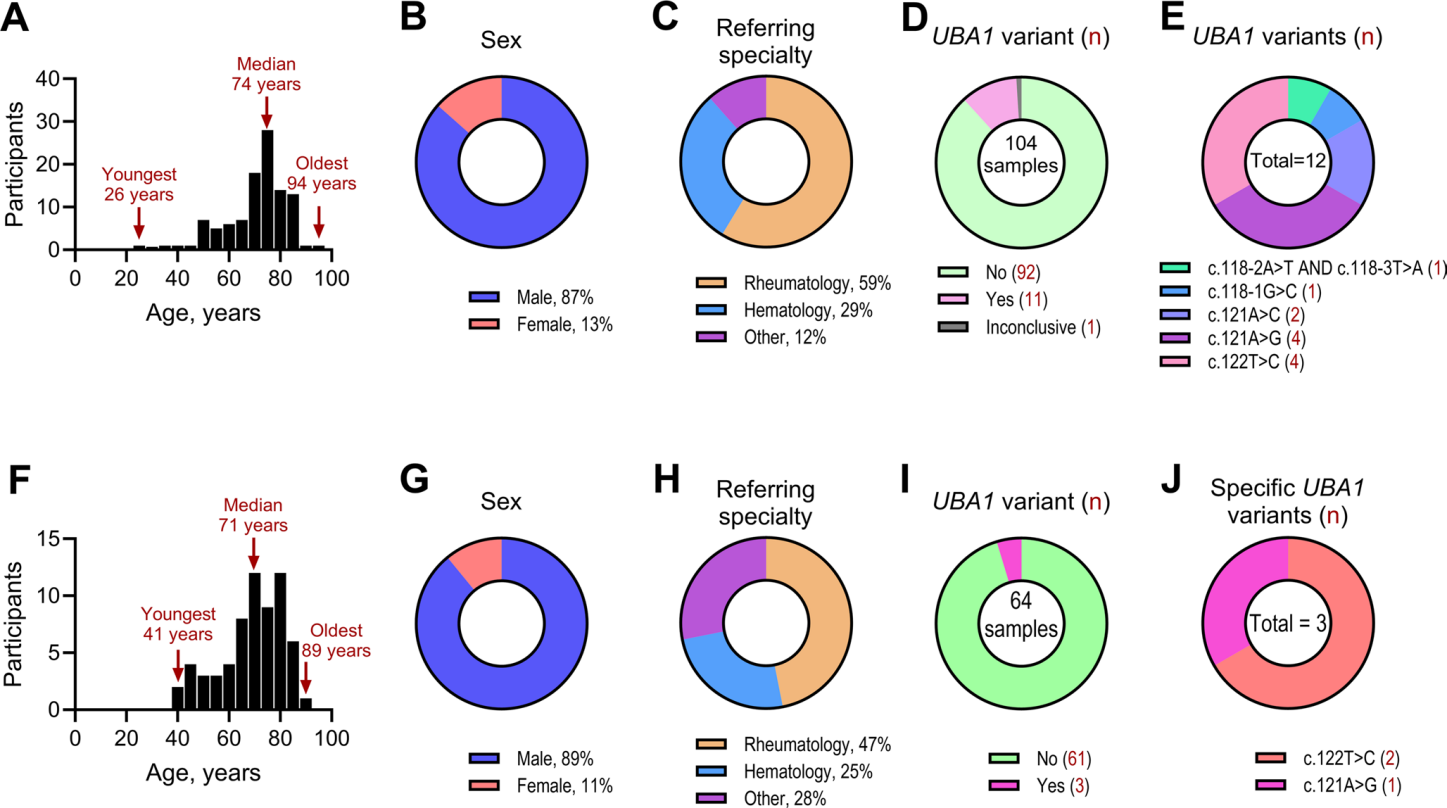
Characteristics of participants **A**–**E**: Demographics of the 104 patients referred for clinical diagnostics of VEXAS-associated *UBA1* variants between March 9, 2021, and October 26, 2023, constituting the primary cohort. (**A**) A histogram depicts the age distribution of participants at the time of testing. (**B**) A donut chart illustrates the sex distribution of the participants. (**C**) A donut chart presents the distribution of referring medical specialties, with ‘Other’ representing various internal medicine departments. (**D**) A donut chart illustrating the proportion of samples identified as positive, negative, or inconclusive for *UBA1* variants based on Sanger sequencing. (**E**) A donut chart displaying the distribution of specific *UBA1* variants detected among the positive samples **F**–**J**: Participants enrolled in the validation cohort, referred for clinical diagnostics of VEXAS-associated UBA1 variants from October 27, 2023, until November 20, 2024, included 64 participants. (**F**) Participants´ median age was 71 years, ranging between 41 years and 89 years. B: Fifty-seven participants were male (89%), and seven were female (11%). (**G**) Participants were referred from tertiary departments of rheumatology (47%) and hematology (25%), whereas the remainder were referred from miscellaneous departments of internal medicine (28%). (**H**) The participants were tested using the Sanger sequencing assay. (**I**) Three participants carried *UBA1* variants, (**J**) one with c.121 A > G and two with c.122T > C

A total of 104 participants were enrolled in the primary cohort from March 9, 2021, until October 26, 2023. The median age was 74 years (range: 26–94), with 90 males (87%), and 14 females (13%) (Fig. 2A and **B**). Participants were primarily referred from rheumatology (59%) and hematology (30%) departments (Fig. 2C). A validation cohort, consisting of 64 participants, was enrolled from October 27, 2023, to November 20, 2024 (Fig. 2F–H).

### Sanger Sequencing-Based Detection of *UBA1* Variants

Sanger sequencing of participants in the primary cohort identified *UBA1* variants in 11 individuals and initially inconclusive results for one (Fig. 2D). The latter participant was retested with two separate samples collected 97 and 102 days after the initial sampling, confirming a *UBA1* variant. Thus, *UBA1* variants were found in 12 participants (12%) in this cohort, all of which were single nucleotide variants (Fig. 2E). In the validation cohort, three participants (4.7%) carried *UBA1* variants (Fig. 2I and **J**).

### NGS-Based Detection of *UBA1* Variants

We developed and validated an amplicon-based NGS assay with a *UBA1* VAF detection limit of 0.0078 (Supplementary Information).

The NGS assay was applied to the primary cohort and confirmed all variants identified by Sanger sequencing. The sample with inconclusive Sanger results was found to contain the c.121 A > G variant (VAF 0.13). Notably, NGS did not detect any true *UBA1* variants in samples classified as negative by Sanger sequencing, though some artifact variants were identified. One sample, which was positive for the c.121 A > G variant (VAF 0.41), contained 11 additional variants distributed across the amplicon, with VAFs ranging from 0.008 to 0.013. This sample was the only one with DNA extracted from a formaldehyde-fixed bone marrow biopsy. The additional 11 variants were categorized as artifacts, presumably caused by the formaldehyde-fixation. This conclusion was based on the observation that individual NGS reads rarely contained more than one of these additional variants, indicating a random process rather than the biologically improbable presence of 12 distinct, low-frequency clones.

In conclusion, NGS confirmed all *UBA1* variants identified by Sanger sequencing, in addition to a few artifacts, thus featuring limited additional diagnostic yield compared with Sanger sequencing.

### Comparison of VAF_*Blood*_ estimation Between NGS and Sanger Sequencing

**Fig. 3:**
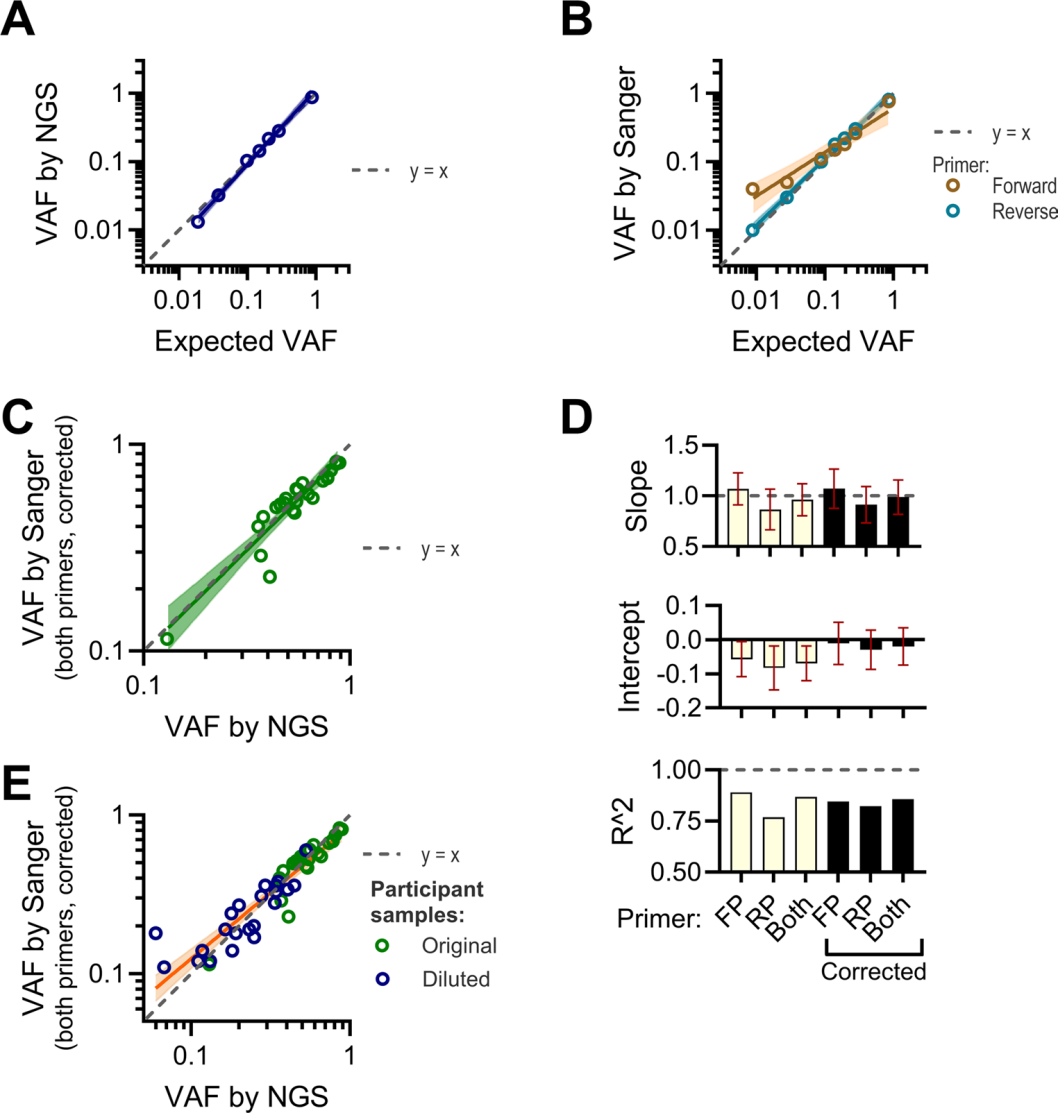
Comparison of VAF estimation between NGS and Sanger sequencing in the primary cohort (**A**) A dilution series was prepared using a DNA sample containing the *UBA1* c.122T > C variant, diluted with a DNA sample lacking *UBA1* variants to samples with expected VAF of 0.86, 0.28, 0.19, 0.14, 0.088, 0.028, and 0.0088. VAFs were determined using the NGS assay with variant calling using mpileup2snp and compared to expected VAFs in an X-Y plot. Regression curve with CI is shown. (**B**) The same dilution series as in panel A was analyzed using Sanger sequencing, with VAFs estimated via EditR. X-Y plots for forward and reverse primers are presented separately, with linear regression curves and CI. (**C**) Linear regression curve with CI comparing VAFs for 27 *UBA1* variant-positive participant samples determined by Sanger sequencing/EditR and NGS, shown in an X-Y plot. Multiple samples, collected at different time points, were included for some participants. (**D**) Bar charts summarizing the results of linear regression analyses of participants’ VAFs determined by NGS and Sanger sequencing/EditR. Data includes forward primer only, reverse primer only, both primers combined, and analyses with or without artifact variant correction. The slopes with CI, intercepts with CI, and R^2 values are displayed at the top, middle, and bottom of the charts, respectively. (**E**) Data from panel C and same analysis but including additional datapoints representing results from 10 samples with *UBA1* variants, diluted with a DNA sample lacking *UBA1* variants to achieve approximately 2-fold and 4-fold lower VAFs

To investigate the accuracy of VAF quantification by NGS and Sanger sequencing, we constructed a dilution series using a sample with the *UBA1* c.122T > C variant diluted with a sample containing the *UBA1* reference sequence. The dilution series was analyzed by both NGS and Sanger sequencing. We found that NGS provided accurate VAFs when compared to the expected VAFs, with a linear regression slope of 1.1 (CI: 1.0–1.2) and an *R*^*2*^ of 1.0 (Fig. 3A). Sanger sequencing was fairly accurate, especially with the reverse primer (Fig. 3B), displaying a slope of 0.98 (CI: 0.91–1.0]) and an *R*^*2*^ of 1.0. The forward primer sequencing data analysis displayed a slope of 0.64 (CI: 0.47–0.82) and an *R*^*2*^ of 0.95. The deviation in the forward primer regression line is due to the overestimation of VAF in the two most diluted samples (expected VAFs of 0.028, and 0.0088).

Next, we analyzed *UBA1* variant-positive samples from participants in the primary cohort by both assays. Six approaches for applying Sanger sequencing data were tested: VAF from forward primer sequencing data, VAF from reverse primer sequencing data, and VAF from averages of sequencing data from both primers, each with and without artifact VAF correction. Artifact VAFs were defined as the summed VAFs at the variant position that did not match the reference or dominant variant nucleotide. For example, for the c.122T > C variant, VAFs reported for A and G nucleotides at position c.122 were categorized as artifacts. Linear regression analyses of logarithmically transformed data showed that all six Sanger-based approaches closely approximated the VAFs determined by NGS, with regression slopes not significantly different from one (Fig. 3C and D). However, intercepts for approaches without artifact VAF correction were significantly below zero (Fig. 3D). Thus, VAFs are underestimated without correction for artifact VAFs. In the remainder of the study, we used sequencing data from both primers with artifact VAF correction.

Figure 3C shows that the participants’ VAFs predominantly fall within the range of 0.4 to 0.9, but it is relevant to determine whether Sanger sequencing can accurately estimate lower VAFs. To investigate this, we constructed samples with approximately 2-fold and 4-fold lower VAFs for 10 participant DNA samples by diluting them using a DNA sample with the *UBA1* reference sequence. These samples were then analyzed using both assays. Inclusion of these datapoints in the regression analysis demonstrated a strong correlation between the two methods, with an *R²* of 0.86 (Fig. 3E). However, the regression line exhibited a slope of 0.83 (CI: 0.73–0.93). This deviation from one appears to be driven by the overestimation of VAFs below 0.1 by Sanger sequencing. Excluding datapoints with NGS-based VAFs below 0.1 improved the correlation between the methods, resulting in an *R²* of 0.91 and a slope of 0.96 (95% CI: 0.87–1.1), which is not statistically significantly different from 1.

To assess the reproducibility of VAF estimations by Sanger sequencing, we re-analyzed three DNA samples, each harboring a distinct *UBA1* variant (c.121 A > C, c.121 A > G, and c.122T > C), on two separate occasions. For the first sample (c.121 A > C), the measured VAFs were 0.29 and 0.40; for the second sample (c.121 A > G), the VAFs were 0.11 and 0.12; and for the third sample (c.122T > C), both measurements yielded a VAF of 0.51. Based on these paired results, we calculated an average coefficient of variation of 10%, suggesting that Sanger-based VAF estimates in our setup are reasonably reproducible.

In conclusion, Sanger sequencing can accurately and reproducibly determine *UBA1* VAF within the range of 0.1 to 0.9.

### VAF in Consecutive Samples from Individual Patients

We quantified VAF using Sanger sequencing in all participants with VEXAS who provided multiple consecutive samples (*n* = 3). The follow-up period ranged from 396 to 1210 days, and the number of samples per patient varied from 10 to 15. In addition to VAF_*Blood*_, we explored the trajectories of two novel parameters: VAF_*Myeloid*_ and VEXAS cell concentration. VAF_*Myeloid*_ was derived from VAF_*Blood*_ by correcting for the proportion of myeloid cells within the leukocyte population. VEXAS cell concentration was estimated by multiplying VAF_*Blood*_ by the total blood leukocyte concentration.

**Fig. 4:**
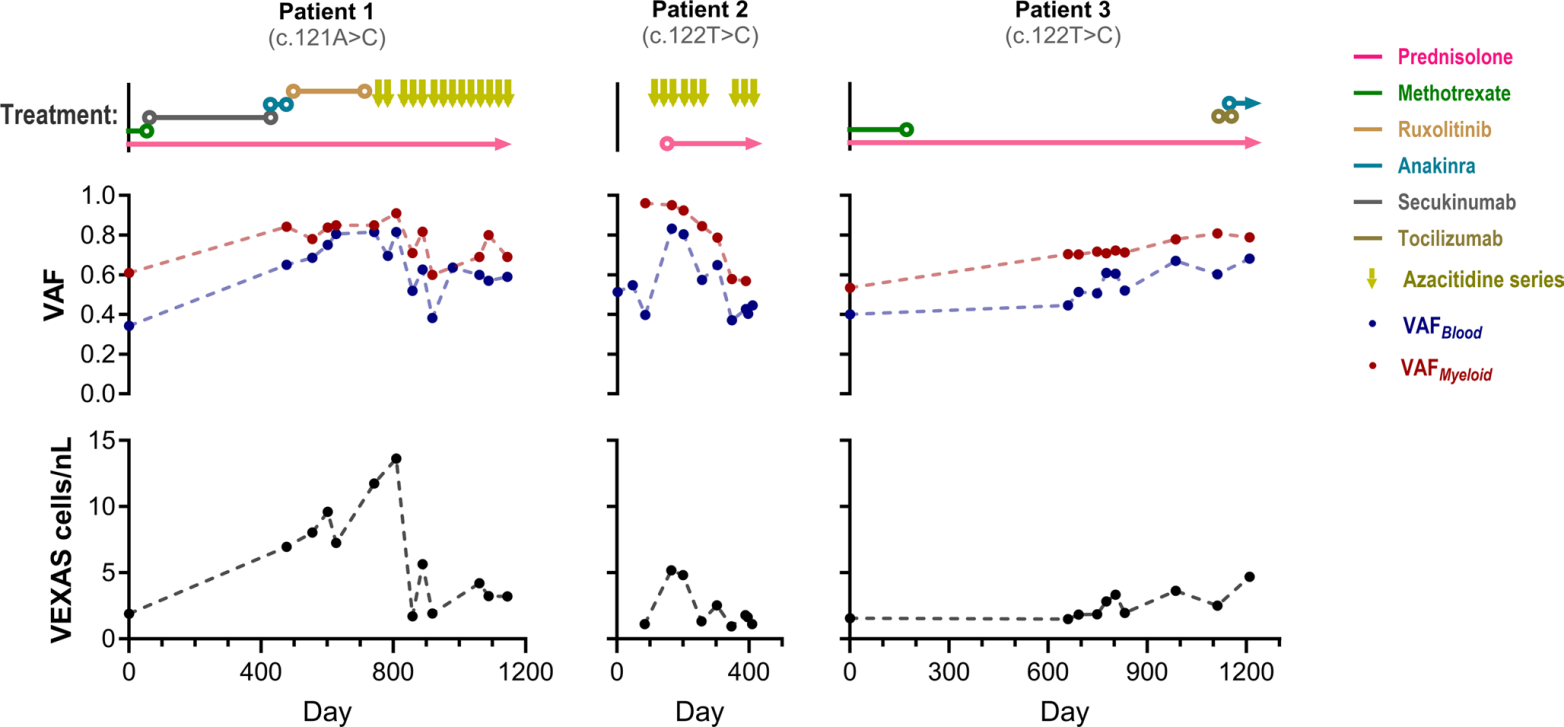
VAF dynamics in sequential patient samples This figure illustrates the temporal changes in VAFs and concentration of VEXAS cells for three individual patients, alongside the corresponding therapeutic interventions they underwent. VAFs were determined through Sanger sequencing, followed by data analysis using EditR. For each patient, VAFs are displayed both as unadjusted estimates from peripheral blood (VAF_*Blood*_) and as corrected values reflecting the proportion within myeloid cells (VAF_*Myeloid*_), under the assumption that VEXAS-associated variants are confined to myeloid cells in the peripheral blood. The concentration of VEXAS cells was calculated by multiplying VAF_*Blood*_ by the leukocyte concentration at the time of testing

VAF_*Blood*_ and VAF_*Myeloid*_ increased over time in patient 1 (prior to Azacitidine treatment) and patient 3 (Fig. 4). In patient 1, Azacitidine treatment was associated with a gradual, irregular decrease in both VAF_*Blood*_ and VAF_*Myeloid*_. In contrast, Azacitidine treatment of patient 2 was accompanied by a continuous, monotonic decline in VAF_*Myeloid*_ whereas the trajectory of VAF_*Blood*_ was less certain.

Comparing VAF_*Blood*_ and VAF_*Myeloid*_ over time, we observed that VAF_*Blood*_ exhibited greater fluctuations from the overall trend in the three patients. This suggests that VAF_*Myeloid*_ may provide a more consistent and reliable indicator of the individual’s allelic burden over time.

During Azacitidine treatment, VEXAS cell concentration in patient 1 decreased dramatically by 76%, from 14/nL to 3.2/nL and by 79% in patient 2, from 5.2/nL to 1.1/nL. These decreases were accompanied by clinical improvement in both patients. Notably, VAF_*Blood*_ decreased only modestly in patient 1 (28%) and actually increased in patient 2 (12%). Patient 3, who exhibited relatively mild symptoms despite VAFs similar to those of the more severely affected patients (1 and 2), generally presented lower VEXAS cell concentrations. Based on these preliminary findings, we propose VEXAS cell concentration as a potential marker of disease burden.

### Correlation of VAFs and VEXAS Cell Concentration with Inflammation in Patients with VEXAS

**Fig. 5:**
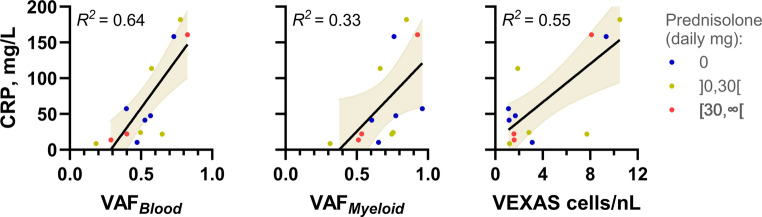
Correlation of VAFs and VEXAS cell concentration with CRP in patients with VEXAS Relationship between CRP levels and the parameters VAF_*Blood*_ (**A**), VAF_*Myeloid*_ (**B**), and VEXAS cell concentration (**C**) in participants with VEXAS and available data for this analysis (*n* = 13). Two participants with VEXAS could not be included: One did not provide a blood sample for testing (only bone marrow), and the other was from a different administrative unit. For the included participants, CRP was measured at the time of *UBA1* variant testing in 11 cases. For the remaining two participants, CRP was not measured at the time of testing; thus, the closest available CRP values were used: one measured six days after *UBA1* testing and the other 20 days before. The daily glucocorticoid dose, expressed in prednisolone-equivalents at the time of testing, is visualized by color for each patient:0 (blue),]0,30 mg [(yellow/green), and 30 mg or more (red)

We investigated the relationship between CRP levels and three parameters—VAF_*Blood*_, VAF_*Myeloid*_, and VEXAS cell concentration—in all participants with VEXAS who had available data for this analysis (*n* = 13). We found that all three parameters positively correlated with CRP levels (Fig. 5).

These results indicate that VAF_*Blood*_, VAF_*Myeloid*_, and VEXAS cell concentration are associated with the degree of inflammation in our participants.

### Association Between Participants´ Characteristics and Test Outcomes

**Fig. 6:**
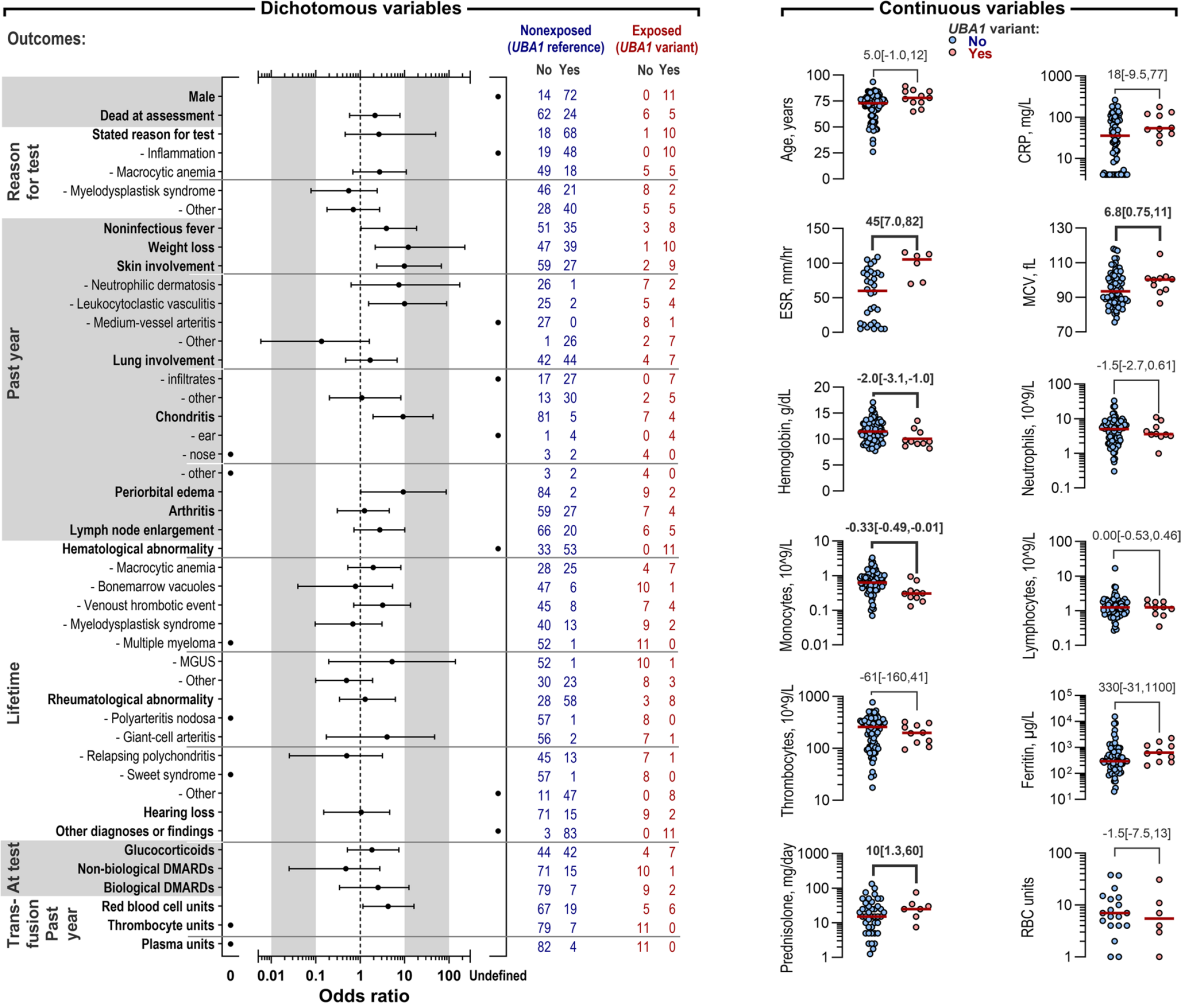
Participant characteristics stratified by *UBA1* variant status. (**A**) Odds ratios with CIs are presented for dichotomous outcome variables, using *UBA1* variant status as the exposure variable. Instances where the odds ratio calculation resulted in a zero denominator are marked as undefined. MGUS: monoclonal gammopathy of undetermined significance. Skin involvement was often described with features of several skin pathologies, leading to some patients being assigned to more than one category of skin involvement. Detailed descriptions of the variables are provided in the Supplementary Material. (**B**) Continuous variables are shown, stratified by *UBA1* variant status. The following abbreviations are used: CRP (C-reactive protein), ESR (erythrocyte sedimentation rate), MCV (mean corpuscular volume of erythrocytes), and RBC units (number of transfused red blood cell units in participants receiving such transfusions). For laboratory parameters, each participant’s median value in the year preceding *UBA1* variant testing was included. The daily glucocorticoid dose, expressed in prednisolone-equivalents was recorded at the time of *UBA1* variant testing, and the number of transfused RBC units corresponds to the year before testing. Medians for each group are indicated by horizontal red bars, and the median difference between groups, along with CIs based on 5,000 bootstrap samples, is displayed in grey text

We investigated the relationship between patient characteristics and *UBA1* variant status in the primary cohort to identify potential markers for VEXAS (Fig. 6). Seven participants were excluded from this analysis as detailed in the Supplementary Information, resulting in a total of 97 participants, 11 of whom carried *UBA1* variants.

In the year before testing, several clinical findings were more common among those with *UBA1* variants: persistent or relapsing noninfectious fever (OR 3.9, CI: 1.0–19), unintended weight loss (OR 12, CI: 2.2–230), any type of skin involvement (OR 9.8, CI: 2.3–67), specifically leukocytoclastic vasculitis (OR 10, CI: 1.5–89), any type of chondritis (OR 9.3, CI: 1.9–44), and periorbital edema (OR 9.3, CI: 1.0–86).

Blood analyses in the year preceding *UBA1* testing revealed that individuals with *UBA1* variants had significantly higher erythrocyte sedimentation rates (median 110 versus 60 mm/hr; median difference, 45 mm/hr; CI: 7.0–82), increased mean erythrocyte corpuscular volume (median 100 versus 94 fL; median difference, 6.8 fL; CI: 0.75–11), lower hemoglobin levels (median 9.5 versus 12 g/dL; median difference, 2.0 g/dL; CI: 1.0–3.1), and lower monocyte levels (median 0.31 versus 0.64 × 10⁹/L; median difference, 0.33 × 10⁹/L; CI: 0.010–0.49) compared to individuals without *UBA1* variants.

Participants with *UBA1* variants who received glucocorticoids were treated with 10 mg/day (CI: 1.3–60) more in prednisolone-equivalent doses compared to those without variants. Likewise, participants with *UBA1* variants received erythrocyte transfusions more often (OR 4.2, CI: 1.2–16) than those without *UBA1* variants.

A history of hematological abnormality was found in all participants with *UBA1* variants compared to 62% of those without variants (*p* = 0.014).

Thus, features such as skin involvement, unintended weight loss, inflammation, and signs of macrocytic anemia were associated with *UBA1* variants in our primary cohort.

### Test of Proposed VEXAS-Scoring Systems

**Fig. 7:**
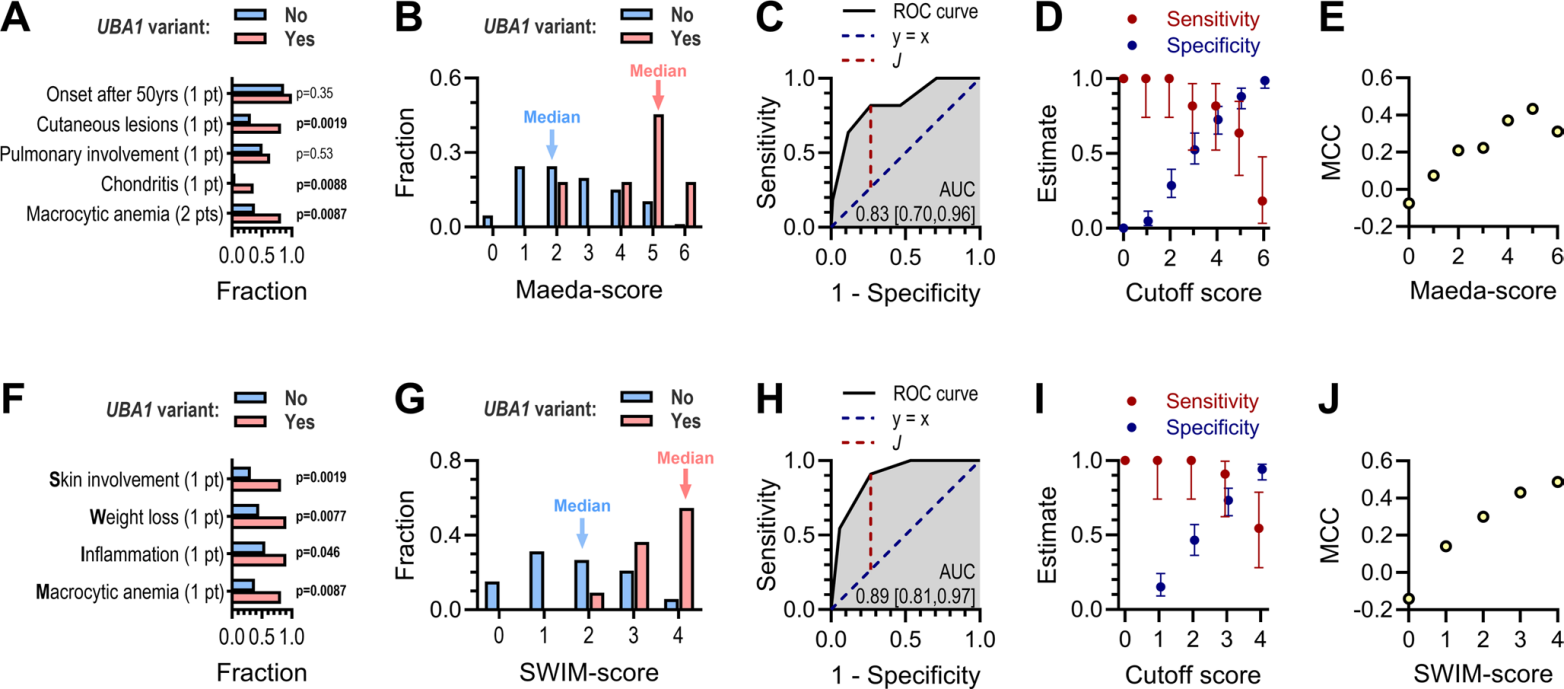
Comparative analysis of VEXAS scoring systems in the primary cohort (**A**) Bar chart showing the proportion of participants, categorized by *UBA1* variant status, who met each criterion in the VEXAS scoring system proposed by Maeda et al. Macrocytic anemia was assigned when stated as reason for testing, documented diagnosis of macrocytic anemia in the medical records, and/or biochemical evidence of macrocytic anemia within the year preceding the test. Statistically significant differences between groups, as determined by Fisher’s exact test, are highlighted in grey. (**B**) Histogram depicting the distribution of Maeda-scores (ranging from 0 to 6) among participants, stratified by *UBA1* variant status. (**C**) ROC curve analysis of the Maeda-score within the cohort. The vertical dotted line marks the score with the highest discriminatory power, identified by Youden’s J statistic (corresponding to a score of 4 or higher). (**D**) Sensitivity and specificity with CIs for Maeda-scores (0 to 6) in the cohort, based on ROC analysis. (**E**) MCC for Maeda-scores (0 to 6) in the cohort. (**F**) Bar chart illustrating the proportion of participants, categorized by *UBA1* variant status, who met each criterion in the proposed SWIM-score system. Macrocytic anemia was assigned the same way as for panel A. (**G**) Histogram showing the distribution of SWIM-scores (ranging from 0 to 4) among participants, stratified by *UBA1* variant status. (**H**) ROC curve analysis of the SWIM-scores within the cohort. The vertical dotted line indicates the score with the highest discriminatory ability, identified by Youden’s J statistic (corresponding to a score of 3 or higher). (**I**) Sensitivity and specificity with CIs for SWIM-scores (0 to 4) in the cohort, derived from ROC analysis. (**J**) MCC for SWIM-scores (0 to 4) in the cohort

We assessed the performance of the Maeda et al. scoring system within the primary cohort. Among the included variables, only cutaneous lesions, chondritis, and macrocytic anemia were significantly more prevalent in participants with *UBA1* variants (Fig. 7A). The median Maeda-score was 2 for participants without *UBA1* variants, compared to 5 for those with *UBA1* variants (Fig. 7B). ROC curve analysis yielded an Area Under the Curve (AUC) of 0.83 (CI: 0.70–0.96) (Fig. 7C). The optimal threshold score, determined by Youden’s *J* statistic, was 4, yielding a sensitivity of 82% (CI: 52–97%) and specificity of 73% (CI: 63–81%) (Fig. 7D). Given the imbalance in our dataset, with 11 participants carrying *UBA1* variants compared to 86 without, the MCC likely provides a more valid measure for determining the optimal threshold score. The highest MCC of 0.43 was observed for a score of 5 (Fig. 7E), corresponding to a sensitivity of 64% (CI: 35–85%) and specificity of 88% (CI: 80–94%). A sensitivity of 100% required a cutoff score of 2, yielding a specificity of 29% (CI: 21–39%) (Fig. 7D).

In summary, the Maeda-score demonstrated optimal performance at higher cutoff levels (4 or preferably 5). Maeda et al. recommend a cutoff score of 3 [9], which would have resulted in two missed VEXAS diagnoses (18%) (Fig. 7B).

To address these limitations in the Maeda-score, we developed an alternative scoring system using data-driven variable selection based on findings from the primary cohort (Fig. 6). We included four variables: **S**kin involvement, Weight loss, Inflammation, and Macrocytic anemia (Fig. 7F), collectively referred to as the SWIM-score. Median SWIM-score was 2 for participants without *UBA1* variants and 4 for those with *UBA1* variants (Fig. 7G). ROC curve analysis yielded an AUC of 0.89 (CI: 0.81–0.97) (Fig. 7H). Youden’s *J* statistic suggested an optimal cutoff of 3, providing 91% sensitivity (CI: 62–100%) and 73% specificity (CI: 63–81%), whereas MCC suggested a cutoff of 4 (Fig. 7I and J). A sensitivity of 100% required a cutoff score of 2, yielding a specificity of 47% (CI: 36–57%), which is statistically significantly higher than the cutoff score required with the Maeda-score for 100% sensitivity.

Both scoring systems were evaluated in a separate validation cohort (Figure S5). The Maeda-score provided an AUC of 0.96 (CI: 0.90–1.0) and the SWIM-score provided an AUC of 0.99 (CI:0.97–1.0). As observed in the primary cohort, the SWIM-score provided significantly higher specificity with a score of 2 compared to the Maeda-score, achieving 65% (CI: 52–76%) versus 42% (CI: 30–54%), respectively.

In conclusion, the simplified SWIM-score demonstrated superior specificity and efficiency compared to the Maeda-score in selecting patients for VEXAS testing.

## Discussion

This study compared Sanger sequencing and amplicon-based NGS for detecting *UBA1* variants in patients with suspected VEXAS, evaluated the use of Sanger sequencing for estimating VAFs, and assessed both the Maeda-score and the simpler SWIM-score for guiding patient selection for *UBA1* testing. We found that NGS and Sanger sequencing had comparable diagnostic yields. Notably, Sanger sequencing effectively determined VAFs within the range of 0.1 to 0.9. The Maeda-score, when used as originally recommended, demonstrated insufficient sensitivity, missing 18% of cases. In contrast, the SWIM-score outperformed the Maeda-score and may be a more reliable tool for selecting patients for genetic testing.

In our cohort of patients with suspected VEXAS who underwent both Sanger sequencing and NGS, no *UBA1* variants were identified by NGS in samples classified as negative by Sanger sequencing, supporting its potential as a cost-effective and more accessible alternative. This finding aligns with previous studies in similar populations [17, 22, 23]. In larger cohorts tested with more sensitive assays, VAFs below the sensitivity threshold of Sanger sequencing, around 0.10, are reported in approximately 0–3% of cases [9, 23, 35]. If Sanger sequencing yields negative results in patients with high suspicion of VEXAS (e.g., SWIM-score ≥ 2), more sensitive testing should be pursued. Such testing should enable low VAF detection in the entire *UBA1* gene as well as other potentially relevant genes.

We found that VAF_*Blood*_, along with VAF_*Myeloid*_ and VEXAS cell concentration, correlated with inflammation as indicated by CRP levels. In contrast, other studies report no correlation between VAF_*Blood*_ and disease severity [20–22]. It seems counterintuitive that no relationship would exist on a population basis if variant-carrying cells drive VEXAS. Small cohort sizes and the lack of standardized definitions of disease severity may explain these diverging findings. However, it is important to note that CRP is a dynamic marker of systemic inflammation, and its levels can fluctuate substantially depending on clinical status, treatment, and intercurrent complications. These limitations should be considered when interpreting the observed correlations and highlight the need for longitudinal studies to better understand the relationship between CRP, VAFs, and disease activity. Nevertheless, at the individual patient level, VAF may offer value for monitoring disease progression and treatment efficacy, as suggested by our study and others [7]. Accurate VAF quantification through Sanger sequencing, as demonstrated in this study, could enhance accessibility to such measurements, thereby facilitating further investigation into its role in clinical monitoring.

Methods for selecting patients for *UBA1* testing are crucial. Given the severity of VEXAS and the low cost of Sanger sequencing, the sensitivity of such methods must approach 100%. We evaluated the Maeda-score and found it to have fair performance; however, Rogez et al. reported significantly lower discriminatory power [10]. Notably, they found that a score of 3, as originally recommended [9], resulted in only 33% sensitivity for early VEXAS [10]. In our primary cohort, a score of 3 would have missed two cases (18%).

These limitations of the Maeda-score can be partly attributed to the inclusion of infrequent and indiscriminatory variables, which likely contribute little to the accuracy of the scoring system. For example, chondritis is present in only a minority of VEXAS patients (present study and Georgin-Lavialle et al. [20]) and may not distinguish those with *UBA1* variants from others, as observed in the cohort used to develop the Maeda-score [9]. Moreover, the unequal weighting of variables complicates clinical use, and such weighting is only justified if the variables provide distinct informative value—something that does not appear to be the case for macrocytic anemia.

To address these issues, we developed the SWIM-score, a simpler, data-driven system with four equally weighted variables: Skin involvement, Weight loss, Inflammation, and Macrocytic anemia. The SWIM-score outperformed the Maeda-score in both our primary cohort and validation cohort.

This study has several strengths, including the rigorous validation of assays and the direct comparison of NGS and Sanger sequencing on identical patient samples. Clinical data were collected prior to test results, minimizing selection bias. By analyzing only clinical data available at the time of testing, our scoring system is directly relevant to real-world decision-making regarding *UBA1* testing. However, the modest sample size and single-center design limit statistical power and generalizability. Our method for estimating VAF_*Myeloid*_ requires future experimental validation. Specifically, the approach would be inappropriate if significant numbers of nucleated, non-leukocyte cells are present in peripheral blood or if significant fractions of lymphocytes carry VEXAS variants. Also, our reliance on data not explicitly collected for the study may underrepresent certain clinical findings, such as bone marrow vacuolization. Additionally, the SWIM-score was developed in a cohort with clinical suspicion of VEXAS, and its generalizability to other populations remains to be determined.

Future research should focus on further validating VAF_*Blood*_, VAF_*Myeloid*_, and VEXAS cell concentration for monitoring disease progression, treatment response, as well as refining scoring systems for selecting patients for *UBA1* testing. Although the SWIM-score showed promise in two cohorts, its validation in external patient populations and diverse clinical settings through multicenter studies is essential for assessing its broader applicability and clinical utility.

In conclusion, Sanger sequencing is a reliable method for detecting *UBA1* variants and their allelic frequencies, with potential for tracking disease progression and treatment response. The SWIM-score has the potential to streamline the diagnostic process for VEXAS, reducing unnecessary testing and associated costs.

## Supplementary Information

Below is the link to the electronic supplementary material.

Supplementary Material 1 (DOCX 507 KB)

## Data Availability

The data supporting the findings of this study are provided within the manuscript and supplementary information or are available upon reasonable request to the corresponding author.
