# Supplementary material for: Diagnostic and Monitoring Strategies for VEXAS Syndrome: Evaluating Sanger Sequencing, NGS, and the SWIM-Score
Source: J Clin Immunol. 2025 Sep 30;45(1):138. doi: 10.1007/s10875-025-01932-9 (PMC12484315; doi:10.1007/s10875-025-01932-9)
Supplement: Supplementary file 1 — Supplementary Material 1 (DOCX 507 KB) [file 10875_2025_1932_MOESM1_ESM.docx]

# **Supplementary Information:** Diagnostic and Monitoring Strategies for VEXAS Syndrome: Evaluating Sanger Sequencing, NGS, and the SWIM-Score


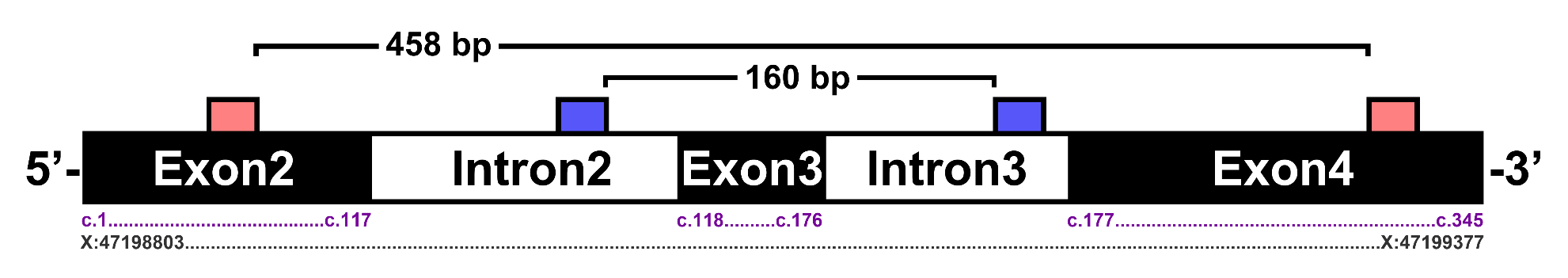


Figure S1: Schematic representation of primer locations in *UBA1* for Sanger sequencing and NGS assay.

Red boxes indicate primer locations used in the Sanger sequencing assay and blue boxes denote primer locations used in the NGS assay.

## Tables

### Table S1

PubMed search string: (vexas) AND (("2020"[Date - Publication] : "2023"[Date - Publication])). Patients clearly presented in more than one study were included only once.

| PMID | Author | Country | Journal | c.118-9_118-2del | c.118-2A>G | c.118-2A>C | c.118-1G>C | c.119 G > C | c.121A>C | c.121A>G | c.121 A > T | c.122T>C | c.167C>T | c.1430G>C | c.1861A>T | Splice mutations' |
| --- | --- | --- | --- | --- | --- | --- | --- | --- | --- | --- | --- | --- | --- | --- | --- | --- |
| 33108101 | Beck DB | USA/UK | N Engl J Med |  |  |  |  |  | 5 | 5 |  | 15 |  |  |  |  |
| 34632574 | Georgin-Lavialle S | France | Br J Dermatol |  |  |  |  |  | 21 | 35 |  | 52 |  |  |  | 8 |
| 33741056 | Huang H | China | Exp Hematol Oncol |  |  |  |  |  | 1 |  |  |  |  |  |  |  |
| 34427584 | Obiorah IE | USA | Blood Adv |  |  |  |  |  | 1 |  |  |  |  |  |  |  |
| 33779074 | Ferrada MA | USA | Arthritis Rheumatol |  |  |  |  |  | 3 | 2 |  | 8 |  |  |  |  |
| 34495287 | Zakine E | France | JAMA Dermatol |  |  |  | 1 |  | 3 | 1 |  | 3 |  |  |  |  |
| 33690815 | Poulter JA | UK | Blood |  |  |  | 1 |  |  | 3 |  | 5 | 1 |  |  |  |
| 34048852 | van der Made | The Netherlands | J Allergy Clin Immunol |  |  |  |  |  | 1 | 4 |  | 7 |  |  |  |  |
| 34817788 | Oo TM | Singapore | J Thromb Thrombolysis |  |  |  |  |  | 1 |  |  |  |  |  |  |  |
| 33789873 | Tsuchida N | Japan | Ann Rheum Dis |  |  |  |  |  | 3 | 2 |  | 3 |  |  |  |  |
| 34805767 | Raaijmakers | The Netherlands | Hemasphere |  |  |  |  |  |  | 1 |  | 2 |  |  |  |  |
| 33987129 | Himmelmann | Schwitzerland | Eur J Case Rep Intern Med |  |  |  |  |  |  | 1 |  |  |  |  |  |  |
| 33460492 | Fan BE | Singapore | Am J Hematol. |  |  |  |  |  | 1 |  |  |  |  |  |  |  |
| 33690844 | Gurnari C | USA | Blood |  |  |  |  |  |  |  |  | 2 |  |  |  |  |
| 34489099 | Koster MJ | USA | Mayo Clin Proc. |  |  |  |  |  | 1 | 1 |  | 7 |  |  |  |  |
| 34864445 | Kao RL | USA | Blood Cells Mol Dis |  |  |  |  |  |  |  |  | 1 |  |  |  |  |
| 34213531 | Templé M | France | Rheumatology (Oxford) | 2 |  |  |  |  |  |  |  |  |  |  |  |  |
| 34046042 | Staels F | Belgium | Front Immunol. |  |  |  |  |  | 1 |  |  | 1 |  |  |  |  |
| 35126364 | Lötscher F | Schwitzerland | Front Immunol |  |  |  |  |  |  |  |  | 1 |  |  |  |  |
| 33881233 | Takahashi N | Japan | Arthritis Rheumatol. |  |  |  |  |  |  | 1 |  |  |  |  |  |  |
| 34463053 | Sharma A | India | Arthritis Rheumatol |  |  |  |  |  |  | 1 |  |  |  |  |  |  |
| 34080084 | Grey A | Australia | J Clin Immunol |  |  |  |  |  |  |  |  | 1 |  |  |  |  |
| 35059089 | Thomas VT | USA | J Hematol |  |  |  |  |  | 1 |  |  |  |  |  |  |  |
| 34391501 | Dehghan N | Canada | Lancet |  |  |  |  |  |  |  |  | 1 |  |  |  |  |
| 34337120 | Alhomida F | USA | JAAD Case Rep. |  |  |  |  |  | 1 |  |  |  |  |  |  |  |
| 33839773 | Sakuma M | Japan | Rheumatology (Oxford) |  |  |  |  |  |  |  |  | 1 |  |  |  |  |
| 34480172 | Shaukat | USA | Mod Rheumatol Case Rep |  |  |  |  |  |  |  |  | 1 |  |  |  |  |
| 34649277 | Li | USA | Blood Adv. |  |  |  |  |  | 1 | 1 |  |  |  |  |  |  |
| 34999727 | Loschi | France | Bone Marrow Transplant |  |  |  |  |  | 1 |  |  |  |  |  |  |  |
| 34714914 | Diarra | France | Blood Adv |  |  |  |  |  | 1 | 4 |  | 1 |  |  |  |  |
| 34587282 | Ribereau-Gayon | France | Int J Dermatol |  |  |  |  |  | 1 |  |  |  |  |  |  |  |
| 35050349 | Beaumesnil | France | JAMA Otolaryngol Head Neck Surg |  |  |  |  |  | 1 | 1 |  | 1 |  |  |  |  |
| 35239266 | Midtvedt | Norway | Tidsskr Nor Laegeforen |  |  |  |  |  |  |  |  | 1 |  |  |  |  |
| 34668539 | Pàmies | Spain | Rheumatology (Oxford) |  |  |  |  |  |  |  |  | 1 |  |  |  |  |
| 35481304 | Ciprian | USA | Cureus |  |  |  |  |  |  | 1 |  |  |  |  |  |  |
| 34611997 | Muratore | Italy | Arthritis Rheumatol |  |  |  |  |  |  | 1 |  | 2 |  |  |  |  |
| 34911285 | Stubbins | Canada | Haematologica |  |  |  |  |  |  |  |  | 1 |  |  |  |  |
| 35419965 | Islam | Australia | Intern Med J |  |  |  |  |  |  | 1 |  | 2 |  |  |  |  |
| 35166394 | Matsubara | Japan | J Dermatol |  |  |  |  |  | 1 |  |  |  |  |  |  |  |
| 35391910 | Goyal | USA | JAAD Case Rep |  |  |  |  |  |  |  |  | 1 |  |  |  |  |
| 35398520 | Matsumoto | Japan | Clin Immunol |  |  |  | 1 |  |  |  |  |  |  |  |  |  |
| 34894213 | Cordts | Germany | Rheumatology (Oxford) |  |  |  |  |  | 1 |  |  |  |  |  |  |  |
| 35597594 | Ronsin | France | Kidney Int |  |  |  | 1 |  |  |  |  |  |  |  |  |  |
| 35950209 | Manzoni | Italy | Clin Hematol Int |  |  |  |  |  |  |  |  | 1 |  |  |  |  |
| 35757758 | Matsumoto | Japan | Front Immunol |  |  |  |  |  |  |  |  | 1 |  |  |  |  |
| 34872982 | Bert-Marcaz | France | J Neurol Neurosurg Psychiatry |  |  |  |  |  | 1 |  |  |  |  |  |  |  |
| 35212178 | Campochiaro | Italy | Arthritis Rheumatol |  |  |  |  |  |  | 2 |  | 1 |  |  |  |  |
| 35238760 | Ciferska | Czech republic | Clin Exp Rheumatol |  |  |  |  |  | 1 |  |  | 2 |  |  |  |  |
| 35302406 | Martín-Nares | Mexico | Scand J Rheumatol |  |  |  |  |  |  | 1 |  |  |  |  |  |  |
| 35366150 | Poulter | UK | J Clin Immunol |  |  |  | 1 |  |  | 3 |  | 2 |  |  |  |  |
| 35094047 | Pathmanathan | Australia | Rheumatology (Oxford) |  |  |  |  |  |  |  |  | 1 |  |  |  |  |
| 35793067 | Grosse | Australia | Med J Aust |  |  |  |  |  |  |  |  | 1 |  |  |  |  |
| 35609174 | Heiblig | USA, France, Portugal | Blood |  |  |  | 2 |  | 8 | 4 |  | 15 |  |  |  |  |
| 36038944 | Wilke | USA | BMC Rheumatol |  |  |  |  |  |  | 1 |  |  |  |  |  |  |
| 36082915 | Gunnarsson | Sweden | Lakartidningen |  |  |  |  |  | 1 |  |  | 1 |  |  |  |  |
| 35793467 | Ferrada MA | USA/UK | Blood |  |  |  |  |  | 15 | 18 |  | 50 |  |  |  |  |
| 36187875 | Gurnari | USA/France/Italy | Hemasphere |  |  |  |  |  |  |  |  | 2 | 1 |  |  |  |
| 35696330 | Itagane | Japan | Arthritis Rheumatol |  |  |  |  |  |  | 1 |  |  |  |  |  |  |
| 35962245 | Yılmaz | Turkey | Clin Rheumatol |  |  |  | 1 |  |  |  |  |  |  |  |  |  |
| 35986821 | Guerrero-Bermúdez | Colombia | Clin Rheumatol |  |  |  |  |  |  | 1 |  |  |  |  |  |  |
| 36039520 | van Leeuwen-Kerkhoff | The Netherlands | Br J Haematol |  |  |  |  |  |  |  |  | 1 |  |  |  |  |
| 36197697 | Nguyen | Australia | Australas J Dermatol |  |  |  |  |  | 1 |  |  | 1 |  |  |  |  |
| 35713495 | Topilow | USA | Rheumatology (Oxford) |  |  |  |  | 1 |  |  | 1 |  |  |  |  |  |
| 35723601 | Shimizu | Japan | Rheumatology (Oxford) |  |  |  |  |  |  |  |  | 1 |  |  |  |  |
| 35924490 | Matsuki | Japan | J Dermatol |  |  |  |  |  |  |  |  | 1 |  |  |  |  |
| 36184391 | Al-Hakim | UK | Br J Haematol |  |  |  |  |  | 1 | 2 |  | 1 |  |  |  |  |
| 36544501 | Tozaki | Japan | Front Med |  |  |  |  |  |  |  |  | 1 |  |  |  |  |
| 36549759 | Neupane | USA | BMJ Case Rep |  |  |  |  |  |  |  |  | 1 |  |  |  |  |
| 35801918 | Lucchino | Italy | Rheumatology (Oxford) |  |  |  |  |  |  | 1 |  |  |  |  |  |  |
| 36252945 | Fagart | France | Clin Nucl Med |  |  |  |  |  | 1 |  |  |  |  |  |  |  |
| 36273795 | Legeas | France | Joint Bone Spine |  |  |  |  |  | 1 |  |  |  |  |  |  |  |
| 35843757 | Holmes | Australia | Pathology |  |  |  |  |  |  |  |  | 1 |  |  |  |  |
| 36403135 | Mangaonkar | USA | Am J Hematol |  |  |  | 1 |  |  | 1 |  | 3 |  |  |  |  |
| 36281520 | Yoon | Korea | Ann Lab Med |  |  |  |  |  | 1 |  |  |  |  |  |  |  |
| 36544349 | Yildirim | Turkey | Int J Rheum Dis |  |  |  |  |  |  | 1 |  |  |  |  |  |  |
| 35230710 | Mohammed | USA | Int J Dermatol |  |  |  | 1 |  |  |  |  | 1 |  |  |  |  |
| 36709502 | Collantes-Rodríguez | Spain | J Dtsch Dermatol Ges |  |  |  |  |  | 1 |  |  |  |  |  |  |  |
| 36264203 | Yamaguchi | Japan | Mod Rheumatol Case Rep |  |  |  |  |  | 1 |  |  |  |  |  |  |  |
| 36742373 | Lucchino | Italy | Rheumatol Adv Pract |  |  |  |  |  |  | 1 |  |  |  |  |  |  |
| 36692560 | Beck | USA | JAMA |  | 1 |  |  |  | 4 | 3 |  | 1 | 2 |  | 1 |  |
| 36735069 | Zeisbrich | Germany | Z Rheumatol |  |  |  |  |  |  |  |  | 1 |  |  |  |  |
| 36879894 | Austestad | Norway | Case Rep Hematol |  |  |  |  |  |  |  |  | 1 |  |  |  |  |
| 36905702 | Battipaglia | Italy | Curr Res Transl Med |  |  |  |  |  |  | 1 |  |  |  |  |  |  |
| 37017017 | Skowron | France | J Eur Acad Dermatol Venereol |  |  |  |  |  |  | 1 |  |  |  |  |  |  |
| 36409014 | Varadarajan | India | QJM |  |  | 1 |  |  |  |  |  |  |  |  |  |  |
| 36690065 | Robert | France | Joint Bone Spine |  |  |  |  |  |  | 1 |  |  |  |  |  |  |
| 37243340 | Bindoli | Italy | Exp Biol Med (Maywood) |  |  |  |  |  | 1 |  |  |  |  |  |  |  |
| 37071935 | R Pinto | Portugal | Acta Med Port |  |  |  |  |  | 4 | 2 |  | 1 |  |  |  |  |
| 36641501 | Kataoka | Japan | Int J Hematol |  |  |  |  |  | 1 |  |  |  |  |  |  |  |
| 37261849 | Fenu | USA | Blood |  |  |  |  |  |  | 1 |  |  |  |  |  |  |
| 37404435 | Estes | USA | Cureus |  |  |  |  |  |  |  |  | 1 |  |  |  |  |
| 36762418 | Stiburkova | Czech Republic | Arthritis Rheumatol |  |  |  |  |  |  |  |  |  |  | 1 |  |  |
| 37062498 | Casal Moura | USA | Respir Med |  |  |  |  |  | 4 | 10 |  | 24 |  |  |  | 7 |
| 37430338 | Vu | USA | BMC Rheumatol |  |  |  |  |  |  |  |  | 1 |  |  |  |  |
| 37084382 | Gutierrez-Rodrigues | USA | Blood |  |  | 1 | 3 |  | 10 | 15 |  | 52 |  |  |  |  |
| 37480098 | Belicard | France | J Med Case Rep |  |  |  |  |  |  | 1 |  |  |  |  |  |  |
| 37521198 | Fahmy | USA | JAAD Case Rep |  |  |  |  |  | 1 |  |  |  |  |  |  |  |
| 37606963 | Maeda | Japan | Rheumatology (Oxford) |  |  |  | 5 |  | 11 | 8 |  | 16 |  |  |  |  |
| 36794903 | Valor-Méndez | Germany | Rheumatology (Oxford) |  |  |  |  |  | 1 |  |  |  |  |  |  |  |
| 37099698 | Karadeniz | Turkey | Rheumatology (Oxford) |  |  |  |  |  | 1 | 1 |  |  |  |  |  |  |
| 37740251 | Sánchez-Hernández | Mexico | Rheumatology (Oxford) |  |  |  |  |  |  |  |  | 1 |  |  |  |  |
| 37287250 | Strasser | Austria | Clin Chem Lab Med |  |  |  |  |  | 1 |  |  |  |  |  |  |  |
| 37062784 | Kunimoto | Japan | Int J Hematol |  |  |  | 1 |  |  |  |  | 1 |  |  |  |  |
| 37073682 | Tosato | Italy | Am J Hematol |  |  |  |  |  |  |  |  | 2 |  |  |  |  |
| 37582690 | Al-Hakim | USA | Br J Haematol |  |  |  |  |  |  |  |  |  | 5 |  |  |  |
| 37595794 | Fanlo | Spain | Arch Soc Esp Oftalmol (Engl Ed) |  |  |  |  |  |  |  |  | 1 |  |  |  |  |
| 37868330 | Pozdniakova | USA | J Med Cases |  |  |  |  |  |  |  |  | 1 |  |  |  |  |
| 37792501 | Oka | Japan | Rheumatology (Oxford) |  |  |  |  |  |  |  |  | 1 |  |  |  |  |
| 37337622 | Salehi | Australia | Int J Rheum Dis |  |  |  |  |  |  | 2 |  | 1 |  |  |  |  |
| 37738165 | Djerbi | Schwitzerland | Blood Adv |  |  |  |  |  |  | 1 |  |  |  |  |  |  |
| 37228016 | Hines | USA | Rheumatology (Oxford) |  |  |  | 1 |  |  | 2 |  | 4 |  |  |  |  |
| 37666646 | Mascaro | Spain | Ann Rheum Dis |  |  |  | 1 |  |  | 6 | 7 | 12 |  |  |  |  |
| 38137719 | Kim | South Korea | J Clin Med |  |  |  |  |  |  |  |  | 1 |  |  |  |  |
| 38108564 | Harrison | USA | Int J Dermatol |  |  |  |  |  | 1 |  |  |  |  |  |  |  |
| 38108589 | De | Indien | Int J Dermatol |  |  |  |  |  |  |  |  | 1 |  |  |  |  |
| 38141211 | Johansen | Denmark | Rheumatology (Oxford) |  |  |  | 1 |  | 2 | 3 |  | 10 |  |  |  |  |
| 37548220 | Miyoshi | Japan | Mod Rheumatol Case Rep |  |  |  | 1 |  |  |  |  |  |  |  |  |  |
| 38206689 | Tsourveloudis | Greece | Medicine (Baltimore) |  |  |  |  |  |  |  |  | 1 |  |  |  |  |
| 38159331 | Kusne | USA | Leuk Res |  |  |  |  |  |  |  |  | 1 |  |  |  |  |
| 38162410 | Wang | USA | JAAD Case Rep |  |  |  |  |  | 1 |  |  |  |  |  |  |  |
| 37674280 | Haines | USA | Int J Lab Hematol |  |  |  |  |  |  | 1 |  |  |  |  |  |  |
| 38108611 | Gurnari | Italy | Am J Hematol |  |  |  | 3 |  | 3 | 11 |  | 21 | 1 | 1 |  |  |

## Methods

### Data collection.

The Danish personal identification number (CPR) served as the identifier for data entry in the database, which was hosted by the Central Denmark Region. CPR numbers were also used to determine the participants’ date of birth (first six digits) and sex (last digit: even for females, odd for males). Blood sampling data were retrieved from the laboratory system at the Department of Clinical Immunology, Aarhus University Hospital. Clinical data were extracted from medical records available only from Aarhus University Hospital and limited to the past five years, in compliance with ethical permissions (see the ethics part of the Material and Methods section in the main article).

#### Data collected for the primary cohort.

A case report form was completed for all patients eligible for inclusion in the primary cohort, ensuring 100% data completeness.

The following general information was collected: status at assessment (if deceased, the date of death), age at onset of symptoms, *UBA1* variant status (if positive, the specific variant), methods used for testing, concentrations of blood leukocytes and lymphocytes at the time of testing, referring department, and the reason for testing as stated in the medical record (including whether this was due to inflammation, macrocytic anemia, myelodysplastic syndrome, and/or other with text specification).

For the year preceding the test, the following dichotomous clinical findings were documented if stated: recurrent or persistent noninfectious fever, weight loss, skin involvement (if present, further classified as neutrophilic dermatosis, leukocytoclastic vasculitis, medium-vessel arteritis, or other, with text specification), lung involvement (if present, further classified as infiltrates and/or other, with text specification), chondritis (if present, specifying the affected sites: ear(s), nose, and/or other, with text specification), periorbital edema, arthritis, and lymph node enlargement.

For the following laboratory findings in peripheral blood, the number and median value of the measurements for the year preceding the test was recorded: erythrocyte sedimentation rate, erythrocyte mean corpuscular volume, and concentrations of C-reactive protein (CRP), neutrophils, lymphocytes, monocytes, hemoglobin, thrombocytes, and ferritin.

Treatment data at the time of testing were also collected, including the use of glucocorticoids (if used, the daily dose in prednisolone equivalents), other non-biological disease-modifying antirheumatic drugs (DMARDs) (if used, the number and text specification), and biological DMARDs (if used, the number and text specification).

Transfusion data from the blood bank laboratory system at Aarhus University Hospital were collected for the year preceding the test, including the number of red blood cell units, thrombocyte units, and plasma units transfused.

Finally, historical data from medical records available for the study were collected. This included past diagnoses and findings such as hematological abnormalities (with specifics on macrocytic anemia, bone marrow vacuoles, venous thrombotic events, myelodysplastic syndrome, multiple myeloma, monoclonal gammopathy of undetermined significance, and/or other with text specification), rheumatological abnormalities (with specifics on polyarteritis nodosa, giant-cell arteritis, relapsing polychondritis, Sweet syndrome, and/or other with text specification), hearing loss, and/or other findings with text specification.

**Performance of VEXAS-scoring systems.** The variable ‘macrocytic anemia’ included macrocytic anemia as a reason for testing, any documented diagnosis of macrocytic anemia in medical records, and/or biochemical evidence of macrocytic anemia within the year preceding the test. Biochemical macrocytic anemia was defined as a median erythrocyte mean corpuscular volume above the internal reference interval (98 fL for individuals over 17 years) combined with a median hemoglobin level below the internal reference interval (7.3 mmol/L for females and 8.3 mmol/L for males) in the year preceding *UBA1* testing.

#### Data collected for the validation cohort.

A case report form was completed for all patients eligible for inclusion in the separate validation cohort, ensuring 100% data completeness.

We specifically gathered information relevant to the tested scoring systems in the year before testing, including: Cutaneous lesions/skin involvement (erythematous to purpuric papules and/or plaques with or without an annular pattern, neutrophilic dermatosis, leukocytoclastic vasculitis, medium-vessel arteritis, or other specified conditions), pulmonary involvement (such as infiltrates or other specified conditions), chondritis (involving the ear, nose, or other specified areas), unintended weight loss, signs of noninfectious inflammation (persistent or relapsing fever and median CRP above 20 mg/L), and macrocytic anemia (simultaneously reduced blood hemoglobin and increased MCV). In addition, we documented whether the age at which the disease or symptoms prompting *UBA1* testing occurred was above or below 50 years

### DNA sequencing

#### Sample collection and DNA extraction

Testing material comprised ethylenediaminetetraacetic acid-stabilized venous peripheral blood or DNA extracted from formaldehyde, paraffin-embedded bone marrow biopsies provided by the Department of Pathology, Aarhus University Hospital. DNA was extracted from 350 µL venous blood using the EZ1&2 DNA Blood 350 µl Kit (QIAGEN, Venlo, Netherlands) on an EZ1 Advanced XL system (QIAGEN) and eluted in 200 µL of elution buffer, according to the manufacturer´s protocol. DNA concentration was measured using the dsDNA Quantification, Broad Range kit (ThermoFisher Scientific, Waltham, MA, USA) on a Qubit 3.0 fluorometer (ThermoFisher Scientific).

#### Polymerase-chain reactions (PCR)

PCR reactions were conducted in 20 µL volumes using the TaKaRa LA PCR^TM^ Kit (TAKARA BIO INC, Kyoto, Japan) on a SensoQuest labcycler (Göttingen, Germany) following standard procedures. Residual single-stranded DNA was removed using ExoSAP-IT (ThermoFisher Scientific).

#### Sequencing

Sequencing was performed at the core facility of the Department of Molecular Medicine, Aarhus University Hospital.

- Sanger sequencing: Conducted using BigDye Terminator Cycle Sequencing on an S1000 Thermal Cycler (Bio-Rad Laboratories, Hercules, Ca, USA). Unincorporated dyes were removed using the CleanSEQ Dye-Terminator Removal Kit (Beckman Coulter, Pasadena, CA, USA) on an automated pipetting robot (Hamilton Robotics, Reno, NV, USA). Sequencing was executed on an Applied Biosystems™ 3500 XL Genetic Analyzer (ThermoFisher Scientific).
- NGS: Index adaptor sequences were added in a second PCR using the Nextera XT DNA Library Preparation Kit (Illumina, San Diego, CA, USA). The product was cleaned with ExoSAP-IT as in the first PCR step, and sequencing was performed as paired-end reads on a Novaseq 6000 (Illumina).

### Sequence analysis – use of GATK HaplotypeCaller

BAM files were processed using GATK HaplotypeCaller (Broad Institute, Cambridge, MA, USA). VCF files were exported from GATK HaplotypeCaller and analyzed using VarSeq version 2.4.0.

### PCR primers.

Sanger sequencing primers (yielding a 498 base pair amplicon):

- Forward: 5’-AAGCCGGGTTCTAACTGCTC-3', targeting chrX: 47198854-47198873 (hg38) in exon 2.
- Reverse: 5’-CAGTGCCCTGGTCATGTAGG-3', targeting chrX: 47199332-47199351 in exon 4.

NGS primers (yielding a 267 base pair amplicon):

- Forward: 5’-GCTCCACTCCTGTGTGTCTC-3', targeting chrX: 47198998-47199017 in intron 2, with an overhang: 5’-TCGTCGGCAGCGTCAGATGTGTATAAGAGACAG-3'.
- Reverse: 5’-GGGGGTACTCTAGGTCAGGG-3', targeting chrX: 47199178-47199197 in intron 3, with an overhang: 5’-GTCTCGTGGGCTCGGAGATGTGTATAAGAGACAG-3'.

### Performance of NGS-assay

We assessed the assay's read depth across the samples, finding a median read depth of 593,187, ranging from 207,922 to 1,734,252 (**Figure S2A**). With this read depth the assay could theoretically detect VAFs as low as 0.0000048 (1/207,922). We evaluated the mpileup2snp processed NGS data for potential artefactual SNVs in the 160 bp amplicon. Notably, all samples contained a C-to-T substitution at position X:47199018, located immediately downstream of the forward primer, with VAF*_Blood_* ranging from 0.12 to 0.20. Given the consistent presence of this variant across all samples and at similar frequencies, we classified it as an artefact and excluded it from further analysis.

To define a lower limit of SNV detection, we examined data for the remaining 159 sequenced positions in 10 random patient samples in more detail. One sample contained a VEXAS variant, c.121A>C, detected by Sanger sequencing, and this variant was also identified in the NGS data with a VAF*_Blood_* of 0.55. Additionally, we identified 711 low-frequency variants in the 10 samples, with VAF*_Blood_* ranging from 0.0012 to 0.0053. Despite appearing to be high-quality calls with a median genotype quality of 255, we found compelling evidence that these represented artefacts:

First, out of the 12 possible nucleotide substitutions, two specific substitutions were highly enriched: A to G (332, 47%) and T to C (317, 43%) (**Figure S2B**). This is unlikely to reflect biology. Second, 71% of all specific low-frequency variants were present in all 10 samples (**Figure S2C**). This is improbable as a coincidence and, therefore, unlikely to reflect biology. None of the low-frequency variants present in only some of the 10 samples were within 10 bp of codon 41, the hotspot for VEXAS variants. Third, we observed a positive correlation between the allele frequency of variants grouped according to nucleotide substitution and the total number of variants with that specific substitution (**Figure S2D**). The relationship between VAF and specific substitutions is likely explained by differing artifact rates associated with those substitutions.

We concluded that these low-frequency variants were likely sequencing artefacts and defined our lower limit of detection as three standard deviations above the highest observed VAF of these artefacts, i.e., 0.0078 for NGS data processed with mpileup2snp.

The detection of insertions and deletions (indels) using mpileup2indel was rare compared to the detection of low-frequency SNVs. We therefore closely examined all positive calls in all analyzed samples. At position X: 47199184, a deletion of ‘C’ was identified in 21% of the samples, with a median VAF of 0.0012, ranging from 0.0011 to 0.0013. Additional deletions were detected: a deletion of ‘CC’ starting at position X:47199017 in one sample (VAF 0.0012), a deletion of ‘A’ at position X:47199021 in another sample, and a deletion of ‘G’ at position X:47199113 in a third sample (VAF 0.0018). The four indels were located in either intron 2 or intron 3. Given their marginal VAFs, we suspected they were artefacts rather than biologically significant variants. Consequently, we did not consider these indels further in our analysis. Notably, GATK HaplotypeCaller did not detect any of these deletions.

###
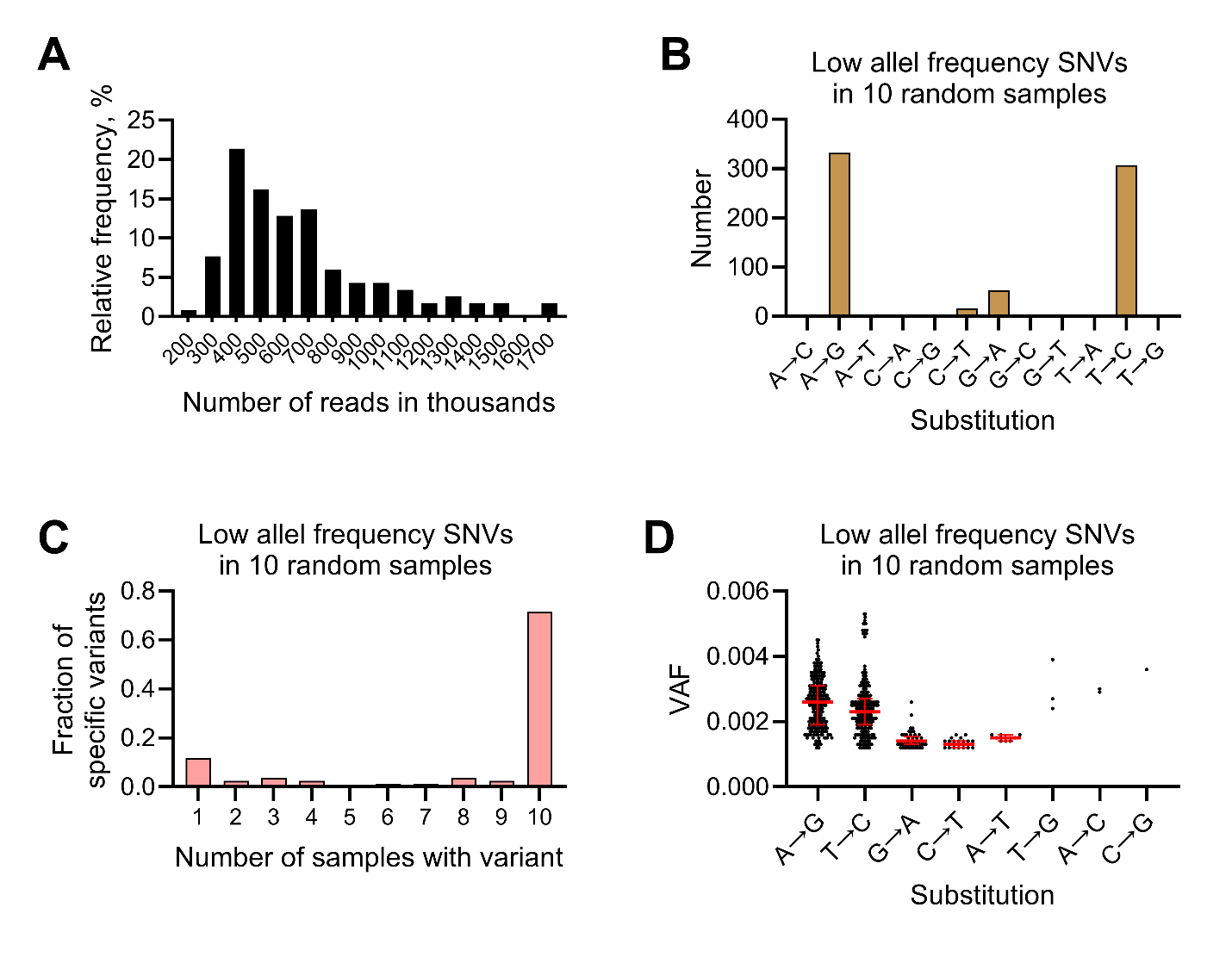


### Figure S2: Amplicon-based NGS detection of *UBA1* variants.

(**A**) Histogram showing the distribution of read depth across samples tested using amplicon-based NGS. (**B**) Bar chart depicting the number of occurrences for each of the 12 possible base substitutions in low-frequency variants (<0.01), detected in ten random samples using SNV calling with mpileup2snp and a cutoff of 0.001. (**C**) Histogram illustrating the distribution of specific low-frequency variants across one to ten samples. (**D**) Scatter plot representing all low-frequency VAFs across the ten samples piled up for each of the 12 possible base substitutions.

### Comparison of VAF*_Blood_* estimation for NGS with different variant callers.

Analyzing NGS data, we found that GATK HaplotypeCaller performed poorly for the low VAF samples, whereas results generated with mpileup2snp closely approximated the expected VAF, with a linear regression slope of 0.95 (95% CI: [0.92, 0.99]) and an *R^2^* of 1.0 (**Figure S3**).

Using GATK HaplotypeCaller on participants´ samples, we identified the same variants as detected by Sanger sequencing, but none of the low-frequency variants identified by mpileup2snp, including the 11 low-abundance variants in the formaldehyde-exposed sample. Additionally, no reliable insertions or deletions were identified with GATK HaplotypeCaller.

###
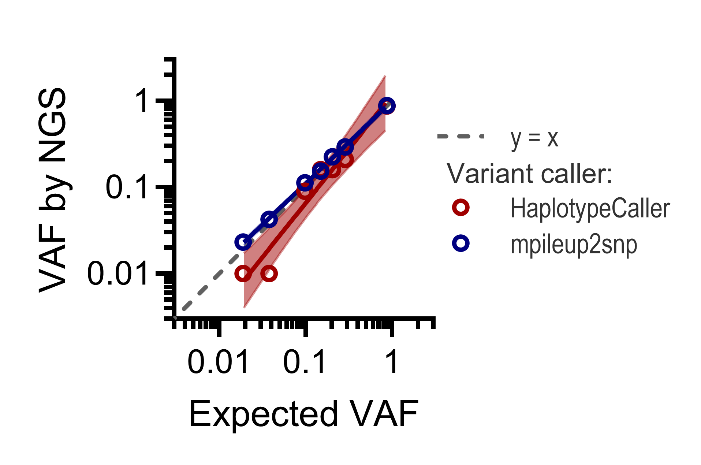


### Figure S3: Comparison of NGS-based VAF estimation with two variant callers.

A dilution series was prepared using a DNA sample containing the *UBA1* c.122T>C variant (VAF of 0.86), which was diluted with DNA lacking UBA1 variants to create samples with expected VAFs of 0.86, 0.28, 0.19, 0.14, 0.088, 0.028, and 0.0088. VAFs were determined using the NGS assay with two different variant callers (HaplotypeCaller and mpileup2snp) and compared to expected VAFs in an X-Y plot. For the two most dilute samples, HaplotypeCaller returned VAF estimates of zero, so data was adjusted by adding 0.01 before logarithmic transformation. Regression curves with 95% CI are shown for both variant callers.


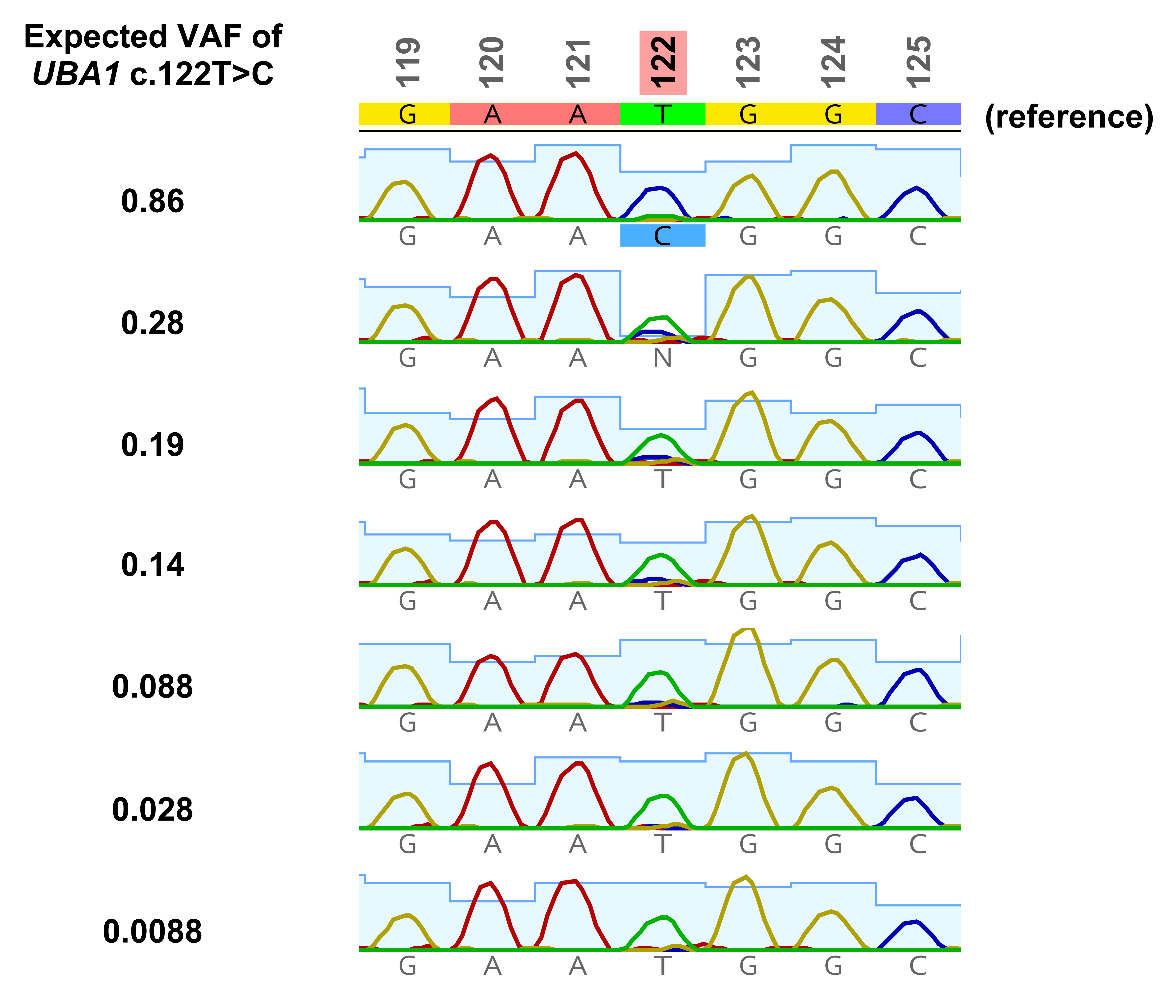


Figure S4: Chromatograms of Sanger sequencing data from a dilution series.

A dilution series was prepared using a DNA sample containing the *UBA1* c.122T>C variant, diluted with a DNA sample containing the *UBA1* reference sequence to achieve samples with expected VAFs of 0.86, 0.28, 0.19, 0.14, 0.088, 0.028, and 0.0088. The samples were analyzed by Sanger sequencing, and the resulting chromatograms are presented.

### Association between participants´ characteristics and test outcomes.

Of the 104 participants in the primary cohort, data for this analysis was unavailable for seven. Five of these participants were from different administrative units, the sixth participant had passed away before the data collection period, and the seventh participant was excluded because he had undergone allogeneic hematopoietic stem cell transplantation with complete engraftment at the time of blood sampling (thus, the sample represented donor DNA rather than the participant’s DNA).

###
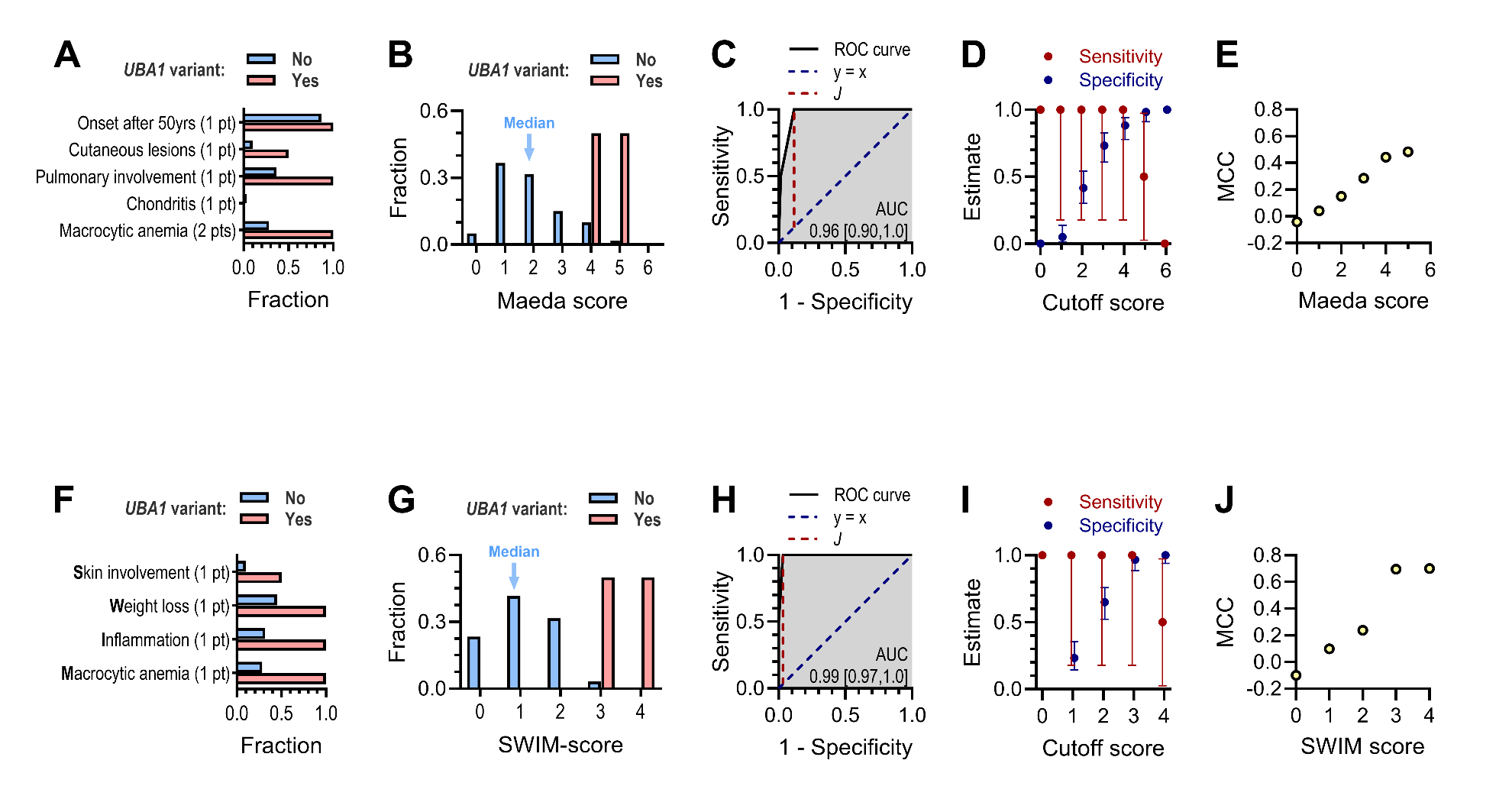


### Figure S5: Comparative analysis of VEXAS scoring systems in the separate validation cohort.

For this analysis, data was available for 62 out of the 64 participants in the validation cohort. Two participants were excluded because they were from external administrative units. One of these excluded participants carried the *UBA1* c.122T>C variant, whereas the other had no identified *UBA1* variants. (**A**) Bar chart showing the proportion of participants, categorized by *UBA1* variant status, who met each criterion in the VEXAS scoring system proposed by Maeda et al. Macrocytic anemia was assigned when stated as reason for testing, documented diagnosis of macrocytic anemia in the medical records, and/or biochemical evidence of macrocytic anemia within the year preceding the test. (**B**) Histogram depicting the distribution of Maeda VEXAS scores (ranging from 0 to 6) among participants, stratified by *UBA1* variant status. (**C**) ROC curve analysis of the Maeda VEXAS score within the cohort. The vertical dotted line marks the score with the highest discriminatory power, identified by Youden's J statistic (corresponding to a score of 4 or higher). Please note that the significantly higher AUC in the validation cohort compared to the primary cohort is likely at least partly due to the more pronounced class imbalance, with an even lower proportion of participants with *UBA1* variants in the validation cohort compared to the primary cohort. (**D**) Sensitivity and specificity with 95% CIs for Maeda scores (0 to 6) in the cohort, based on ROC analysis. (**E**) MCC for Maeda scores (0 to 6) in the cohort. (**F**) Bar chart illustrating the proportion of participants, categorized by *UBA1* variant status, who met each criterion in the proposed SWIM-score system. Macrocytic anemia was assigned in the same manner as described for panel A. (**G**) Histogram showing the distribution of SWIM-scores (ranging from 0 to 4) among participants, stratified by *UBA1* variant status. (**H**) ROC curve analysis of the SWIM-scores within the cohort. The vertical dotted line indicates the score with the highest discriminatory ability, identified by Youden's J statistic (corresponding to a score of 3 or higher). (**I**) Sensitivity and specificity with 95% CIs for SWIM-scores (0 to 4) in the cohort, derived from ROC analysis. (**J**) MCC for SWIM-scores (0 to 4) in the cohort.
